# Association of moderate and vigorous physical activity with incidence of type 2 diabetes and subsequent mortality: 27 year follow-up of the Whitehall II study

**DOI:** 10.1007/s00125-019-05050-1

**Published:** 2019-12-02

**Authors:** Manasa S. Yerramalla, Aurore Fayosse, Aline Dugravot, Adam G. Tabak, Mika Kivimäki, Archana Singh-Manoux, Séverine Sabia

**Affiliations:** 1 Inserm U1153, CRESS, Epidemiology of Ageing and Neurodegenerative Diseases, Université de Paris, 75010 Paris, France; 2 Department of Epidemiology and Public Health, University College London, London, UK; 3 1st Department of Medicine, Semmelweis University, Faculty of Medicine, Budapest, Hungary

**Keywords:** All-cause mortality, Cardiovascular disease mortality, Incident type 2 diabetes, Longitudinal cohort, Long-term association, Moderate-to-vigorous physical activity

## Abstract

**Aims/hypothesis:**

This work examined the role of physical activity in the course of diabetes using data spanning nearly three decades. Our first aim was to examine the long-term association of moderate and vigorous physical activity with incidence of type 2 diabetes. Our second aim was to investigate the association of moderate-to-vigorous physical activity post-diabetes diagnosis with subsequent risk of all-cause and cardiovascular disease mortality.

**Methods:**

A total of 9987 participants from the Whitehall II cohort study free of type 2 diabetes at baseline (1985–1988) were followed for incidence of type 2 diabetes, based on clinical assessments between 1985 and 2016 and linkage to electronic health records up to 31 March 2017. We first examined the association of moderate and vigorous physical activity measured by questionnaire in 1985–1988 (mean age 44.9 [SD 6.0] years; women, 32.7%) with incident type 2 diabetes, using the interval-censored, illness–death model, a competing risk analysis that takes into account both competing risk of death and intermittent ascertainment of diabetes due to reliance on data collection cycles (interval-censored). The second analysis was based on individuals with type 2 diabetes over the follow-up period where we used Cox regression with inverse probability weighting to examine the association of moderate-to-vigorous physical activity after diagnosis of type 2 diabetes with risk of all-cause and cardiovascular disease mortality.

**Results:**

Of the 9987 participants, 1553 developed type 2 diabetes during a mean follow-up of 27.1 (SD 6.3) years. Compared with participants who were inactive in 1985–1988, those who undertook any duration of moderate-to-vigorous physical activity had a lower risk of type 2 diabetes (HR 0.85 [95% CI 0.75, 0.97], *p* = 0.02; analysis adjusted for sociodemographic, behavioural and health-related factors). In 1026 participants with a diagnosis of type 2 diabetes over the follow-up period, data on moderate-to-vigorous physical activity after diabetes diagnosis were available; 165 all-cause deaths and 55 cardiovascular disease-related deaths were recorded during a mean follow-up of 8.8 (SD 6.1) years. In these participants with diabetes, any duration of moderate-to-vigorous physical activity was associated with lower all-cause mortality (HR 0.61 [95% CI 0.41, 0.93], *p* = 0.02) while the association with cardiovascular mortality was evident only for physical activity undertaken at or above recommendations (≥2.5 h per week of moderate-to-vigorous physical activity or ≥1.25 h per week of vigorous physical activity; HR 0.40 [95% CI 0.16, 0.96], *p* = 0.04) in fully adjusted models.

**Conclusions/interpretation:**

Moderate-to-vigorous physical activity plays an important role in diabetes, influencing both its incidence and prognosis. A protective effect on incidence was seen for durations of activity below recommendations and a marginal additional benefit was observed at higher durations. Among individuals with type 2 diabetes, any duration of moderate-to-vigorous physical activity was associated with reduced all-cause mortality while recommended durations of physical activity were required for protection against cardiovascular disease-related mortality.

**Data availability:**

Whitehall II data, protocols and other metadata are available to the scientific community. Please refer to the Whitehall II data sharing policy at https://www.ucl.ac.uk/epidemiology-health-care/research/epidemiology-and-public-health/research/whitehall-ii/data-sharing.

**Electronic supplementary material:**

The online version of this article (10.1007/s00125-019-05050-1) contains peer-reviewed but unedited supplementary material, which is available to authorised users.

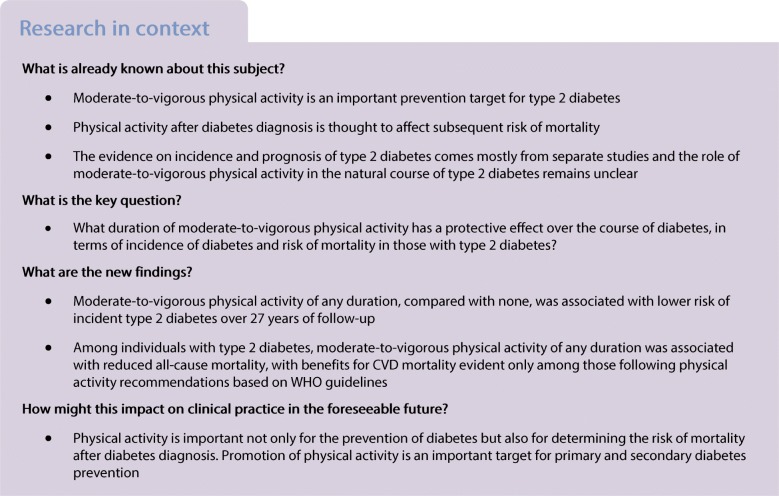



## Introduction

The International Diabetes Federation estimated that 425 million people worldwide had diabetes mellitus in 2017 and this number is expected to reach 629 million by 2045 [1]. Diabetes increases the risk of cardiovascular disease (CVD) and adversely affects survival rate [2, 3]. Given the morbidity and mortality burden of diabetes [1], it is important to identify modifiable factors that affect the natural course of the disease, in terms of development of diabetes and subsequent risk of mortality.

Physical activity is an important target for the prevention of type 2 diabetes [4], the most common form of diabetes [5–7]. However, given the heterogeneity in physical activity assessment in previous studies [7–9] it is important to identify the duration and intensity of physical activity required to confer protection against incidence of diabetes and provide evidence for specific recommendations on physical activity [7]. Some studies have shown moderate-to-vigorous physical activity (MVPA) to be associated with lower incidence of type 2 diabetes [10–12]; one study examined the shape of the association and showed that any duration of MVPA reduced the risk [11]. In addition, type 2 diabetes diagnosis in these studies was obtained from interval-censored data (i.e. intermittent rather than continuous assessments over follow-up) but this feature was rarely addressed in the analyses. These studies may have produced biased results as they did not address the competing risk of death that occurred between two cycles of diabetes ascertainment [13]. Finally, most previous studies had a mean follow-up of less than 10 years and thus were uninformative on the potential long-term protective effect of physical activity on type 2 diabetes incidence [7, 14].

Observational prognostic studies, where people with diabetes are mainly recruited from populations of patients [15] or diabetes is identified at the inception of a general population cohort [16, 17], also show that physical activity among individuals with type 2 diabetes reduces the risk of all-cause mortality [15–18], CVD incidence [18, 19] and CVD mortality [16, 17, 19]. The evidence from randomised controlled trials is mixed; there is robust evidence of a protective effect of lifestyle interventions to delay or reduce the risk of type 2 diabetes [20–22]. However, in people with type 2 diabetes, lifestyle interventions do not show reduced risk for all-cause mortality [23] or CVD outcomes [24]. Most interventions are multi-domain, making it difficult to dissect the specific role of physical activity.

The role of MVPA in the natural course of diabetes, from a heathy population to incidence of type 2 diabetes and subsequent risk of all-cause or CVD-related mortality, has not yet been examined in a single analytical setting. This study has the following aims: (1) to assess the long-term association of MVPA with incident type 2 diabetes over nearly three decades, accounting for interval-censored data and competing risk of death; and (2) to investigate whether MVPA after type 2 diabetes diagnosis is associated with the risk of all-cause and CVD-related mortality.

## Methods

### Study population

The Whitehall II study is an ongoing prospective cohort study established in 1985–1988 among 10,308 London-based civil servants (67% men) aged 35–55 years at inclusion [25]. Baseline assessment (1985–1988) consisted of a questionnaire and a structured clinical evaluation composed of measures of anthropometric, cardiovascular and metabolic risk factors and diseases. Subsequent clinical examinations took place approximately every 4–5 years since baseline, with the latest one completed in 2015–2016. Informed written consent was provided by the participants. The research ethics approvals were renewed at each contact; the latest approval was made by the Joint UCL/UCLH Committee on the Ethics of Human Research, reference number 85/0938.

### Physical activity assessment

Over the follow-up from 1985–1988 to 2015–2016, physical activity was assessed seven times using a questionnaire; these measures of physical activity have been shown to be associated with cardiometabolic outcomes in the Whitehall II cohort study [26–29]. Data on physical activity were drawn from 1985–1988 for the first aim and from the first available wave after diabetes diagnosis for the second aim. During the 1985–1988 and 1991–1993 assessments, participants were asked about the duration and frequency of participation in mildly energetic (e.g. weeding, general housework, bicycle repair), moderately energetic (e.g. dancing, cycling, leisurely swimming) and vigorous physical activity (e.g. running, hard swimming, playing squash) by giving examples for each intensity of physical activity. Starting from the 1997–1999 wave, the questionnaire was modified to reflect the validated Minnesota leisure-time physical activity questionnaire [30] and included 20 items on the frequency and duration of various types of physical activity (e.g. walking, cycling, sports). The participants were required to provide the total number of hours spent doing that activity over the previous 4 weeks for each of the 20 items. We assigned a metabolic equivalent (MET) value to each activity by using a compendium of activity energy costs. One MET is defined as the resting metabolic rate obtained when sitting quietly [31]. An activity with a MET value of 3–5.9 was coded as moderate physical activity (MPA) and an activity with a MET value ≥6 was considered vigorous physical activity (VPA) [32, 33]. The current physical activity guidelines recommend adults to undertake at least 150 min (2.5 h) of MPA per week or 75 min (1.25 h) of VPA per week, or an equivalent combination of moderate and vigorous intensity activity [34]. Time spent in MVPA was calculated as the sum of time spent in MPA and VPA. Values were recorded in decimal point from 1991–1993 assessments, to allow for a more precise categorisation of the duration.

### Ascertainment of type 2 diabetes

We identified individuals with type 2 diabetes using data from clinical assessments between 1985 and 2016 and linkage to the national Hospital Episode Statistics (HES) database (until 31 March 2017) which was undertaken using the National Health Service (NHS) identification number. The following diagnostic criteria were used [35]: fasting glucose ≥7.0 mmol/l (126 mg/dl), use of diabetes medication, reported physician-diagnosed diabetes or HES record of diabetes (ICD-9 code 250 [www.icd9data.com/2007/Volume1] or ICD-10 code E11 [http://apps.who.int/classifications/icd10/browse/2016/en]). Participants with at least one of these criteria were considered to have type 2 diabetes.

### Mortality outcome

Mortality data were collected until 31 March 2017 from the British national mortality register (NHS Central Register). The NHS identification number of each participant was used to carry out the tracing exercise. Deaths related to CVD were identified using ICD codes for CHD (ICD-9 codes 410–414, ICD-10 codes I20–I25) and stroke (ICD-9 codes 430, 431, 434, 436, ICD-10 codes I60–I64).

### Covariates

For the first aim, covariate data were drawn from 1985–1988 and for the second aim from the first available wave after diabetes diagnosis. Sociodemographic variables included age, sex, ethnicity (white/non-white), marital status (married/cohabitating vs others), education (less than primary school [up to age 11], lower secondary school [up to age 16], higher secondary school [up to age 18], university, higher university degree) and occupational position (administrative, professional/executive, clerical/support). Behavioural factors included smoking (never, past, current), alcohol consumption (none, 1–14 units/week, >14 units/week), and fruits and vegetables consumption (< once a day, once a day, ≥ twice daily). BMI was calculated using the measures of weight and height at the clinical examination and categorised as <20, 20–24.9, 25–29.9, and ≥30 kg/m^2^. Starting from 1991–1993, the 36-Item Short Form Health Survey (SF-36) was used to assess general health and quality of life, from which the physical component summary (PCS) and mental component summary (MCS) were derived and included in the analysis [36].

Cardiovascular health-related factors comprised hypertension (systolic/diastolic BP ≥140/90 mmHg, or use of antihypertensive medications) and total cholesterol assessed at the clinical examination, self-reported use of CVD drugs (antiplatelet, diuretic, antihypertensive, lipid-lowering and anticoagulant medication, and β-blockers), and prevalent CVD, including CHD identified by study-specific assessments (12-lead resting ECG recording coded using the Minnesota system) and linkage to HES database (ICD-9 codes 410–414, ICD-10 codes I20–I25, or procedures K40-K49, K50, K75, U19) and stroke determined using linkage to HES database (ICD-9 codes 430, 431, 434, 436 and ICD-10 codes I60–I64).

### Statistical analysis

We conducted two sets of analyses, corresponding to the two aims. We tested the differences in the risk factors by categories of MVPA and diabetes status, separately by using χ^2^ test, student’s *t* test and one way ANOVA. In all analyses, a two-sided *p* < 0.05 was considered statistically significant and the exact *p* value associated with each HR was provided to allow consideration for multiple testing.

#### Role of MVPA in the aetiology of diabetes

The shape of the associations of MPA, VPA and MVPA (continuous variables) assessed at baseline (1985–1988) with incident diabetes was examined using restricted cubic spline regressions with Harrell knots [37] in Cox regression models. The proportionality assumption was assessed using Schoenfeld’s test. Then, time spent in MPA was categorised as none (0 h/week), 1, 2, 3–4 and ≥5 h/week. Time spent in VPA was categorised as none (0 h/week), 1 and ≥2 h/week. Alternative categorisations were also used: physically active (>0 h/week of MVPA) vs inactive (0 h/week of MVPA); following physical activity recommendations (≥3 h/week of MVPA or ≥2 h/week of VPA) vs not following recommendations. The cut-off for recommended duration was ≥3 h instead of ≥2.5 h for MVPA and ≥2 h instead of ≥1.25 h for VPA [34] as the amount of time spent in physical activity was recorded as a whole number in 1985–1988. Interactions of physical activity measures with sex and age were also tested.

Date of type 2 diabetes diagnosis corresponded to the first record of type 2 diabetes using data from all sources. As fasting glucose was a key component of diabetes diagnosis, the exact date of diabetes onset was not known as it depended on the dates of clinical examination undertaken every 4–5 years (interval-censored data). Furthermore, some participants could have developed diabetes and died in between two clinical examinations, making death a competing event. We used an interval-censored, illness–death model, a competing risk analysis which takes into account the interval-censored nature of the data, to estimate transitions between three states (diabetes-free, diabetes and death) [13, 38]. A Weibull distribution was used as it provides a good fit for chronic disease incidence and mortality rates [39].

Analyses were first unadjusted, then age was used as time-scale and models were adjusted for sociodemographic and behavioural factors as well as mutually adjusted for MPA and VPA in analyses on these physical activity variables, then further adjusted for BMI, and finally for cardiovascular health-related variables. In sensitivity analysis, missing data on physical activity (*n* = 155) and covariates (*n* = 66) at baseline were imputed using chained equation modelling and data were reanalysed including these participants.

#### Role of post-diabetes MVPA in subsequent risk of all-cause and CVD mortality

These analyses were based on participants who developed type 2 diabetes during the follow-up and had physical activity measurement and other covariates assessed at the first available wave of data after type 2 diabetes diagnosis. A Cox proportional hazard model was used to assess the association of physical activity after diabetes diagnosis with risk of subsequent all-cause and CVD mortality, separately. Inverse probability weighting (IPW) was used to account for missing data [40]. This involved calculation of the probability of being included in the analytical sample using data on MVPA (four categories: 0, 1–2, 3–4 and ≥5 h of MVPA per week), sociodemographic variables, behavioural variables, BMI, hypertension, total cholesterol, self-reported use of CVD drugs in 1985–1988, chronic conditions (chronic obstructive pulmonary disease, cancer, CVD), and mortality status over the follow-up, including an interaction between mortality status and MVPA categories. The inverse of these probabilities were used to weight the results from Cox regression.

Follow-up time for CVD mortality was from the date of physical activity measurement post-diabetes diagnosis until the date of CVD-based death, non-CVD-based death or end of follow-up (31 March 2017), whichever came first. Restricted cubic spline regressions with Harrell knots [37] were used to examine the shape of the associations of MPA, VPA and MVPA with risk of all-cause and CVD mortality. Given the small number of participants undertaking VPA and the number of deaths in this group, further analyses were undertaken for MVPA categorised as follows: inactive (0 h/week of MVPA), below recommendations (0.1–2.4 h/week of MVPA and <1.25 h/week of VPA) and following recommendations (≥2.5 h/week of MVPA or ≥1.25 h/week of VPA).

Analyses were first unadjusted, then age was used as time-scale and models sequentially adjusted for the time between diabetes diagnosis and MVPA assessment, sociodemographic and behavioural factors, then further for BMI and, finally, for health-related factors (hypertension, total cholesterol, CVD drugs, prevalent CVD and two general health measures, the SF-36 physical and mental health component summary scores, to account for their potential confounding effect [41]). The proportionality assumption was assessed using Schoenfeld’s test.

Three sensitivity analyses were undertaken to assess the validity of the results. First, to ensure that variability in time between diabetes diagnosis and MVPA assessment did not affect results, we re-ran the analysis excluding data where the interval was greater than 6 years. Second, we excluded participants with prevalent CVD at physical activity measurement when CVD mortality was the outcome. Third, we used multiple imputation instead of IPW to account for missing data.

### Software

Analyses were conducted using Stata software (release 15.1; StataCorp, College Station, TX, USA), apart from the interval-censored, illness–death model, which was undertaken using R version 3.5.0 (https://cran.r-project.org/) SmoothHazard package [38].

## Results

### Role of MVPA in the aetiology of diabetes

Of 10,308 participants at baseline, 100 had prevalent diabetes, 155 had missing data on physical activity and 66 had missing data on other covariates, and were excluded from the analyses. Our analyses were therefore based on 9987 participants (see flowchart in electronic supplementary material [ESM] Fig. 1), among whom 1553 developed diabetes during a mean follow-up of 27.1 (SD 6.3) years. Compared with participants included in the analytical sample (*n* = 9987), those excluded due to missing data (*n* = 221) were older (excluded vs included participants: 46.6 vs 44.9 years, *p* < 0.001), more likely to be women (51.1% vs 32.7%, *p* < 0.001) and were from a lower occupational position (53.9 vs 21.8%, *p* < 0.001) but did not differ in terms of incident diabetes (18.1 vs 15.5%, *p* = 0.30).

At baseline (1985–1988), participants undertook, on average, 3.8 (SD 4.2) h/week of MVPA. Mean weekly MVPA duration decreased by 0.2 h (95% CI, 0.1, 0.3) by 2002–2004 (mid follow-up) and by 0.6 h (95% CI 0.5, 0.7) at the last visit before the censoring date (diabetes date, death or March 2017). Participants who met physical activity recommendations were more likely to be white, male, married/cohabitating, from higher socioeconomic background, non-smokers, to drink >14 alcohol units/week, to eat fruits and vegetables more frequently and to have a BMI <30 kg/m^2^ (all *p* < 0.05; Table 1). Participants who developed diabetes were more likely to be non-white, from a lower socioeconomic background, smokers, to eat fruits and vegetables less than daily and have a worse cardiometabolic profile (all *p* < 0.05; Table 1).

**Table 1:** Sample characteristics in 1985–1988 (*N* = 9987)

Characteristic	Total population^a^	MVPA^b^				Diabetes status		
		Inactive (no MVPA)	Below recommendations	Following recommendations	*p* value	No diabetes	Diabetes	*p* value
*N* (row %)	9987	1607 (16.1)	3006 (30.1)	5374 (53.8)		8434 (84.5)	1553 (15.6)	
Mean (SD) age, years	44.9 (6.1)	46.5 (6.0)	44.7 (6.1)	44.5 (6.0)	<0.001	44.7 (6.0)	45.8 (6.0)	<0.001
Female sex	3263 (32.7)	922 (57.4)	1050 (34.9)	1291 (24.0)	<0.001	2762 (32.8)	501 (32.3)	0.706
Non-white	1038 (10.4)	344 (21.4)	281 (9.4)	413 (7.7)	<0.001	715 (8.5)	323 (20.8)	<0.001
Married/cohabiting	7410 (74.2)	1031 (64.2)	2191 (72.9)	4188 (77.9)	<0.001	6255 (74.2)	1155 (74.4)	0.863
University degree or higher	2600 (26.0)	256 (15.9)	875 (29.1)	1469 (27.3)	<0.001	2256 (26.8)	344 (22.2)	<0.001
Low occupational position	2180 (21.8)	804 (50.0)	552 (18.4)	824 (15.3)	<0.001	1723 (20.4)	457 (29.4)	<0.001
Current smoker	1820 (18.2)	404 (25.1)	509 (16.9)	907 (16.9)	<0.001	1499 (17.8)	321 (20.7)	0.007
Consumes >14 units of alcohol per week	2389 (23.9)	263 (16.4)	681 (22.7)	1445 (26.9)	<0.001	2050 (24.3)	339 (21.8)	0.035
Consumes fruits and vegetables less than daily	4174 (41.8)	767 (47.7)	1257 (41.8)	2150 (40.0)	<0.001	3482 (41.3)	692 (44.6)	0.016
BMI ≥30.0 kg/m^2^	686 (6.9)	188 (11.7)	192 (6.4)	306 (5.7)	<0.001	412 (4.9)	274 (17.6)	<0.001
Hypertension	1884 (18.9)	325 (20.2)	592 (19.7)	967 (18.0)	0.051	1484 (17.6)	400 (25.8)	<0.001
Mean (SD) total cholesterol, mmol/l	6.0 (1.2)	6.1 (1.2)	5.9 (1.2)	5.9 (1.1)	<0.001	5.9 (1.2)	6.2 (1.2)	<0.001
CVD drugs	322 (3.2)	70 (4.4)	115 (3.8)	137 (2.6)	<0.001	252 (3.0)	70 (4.5)	0.003
Prevalent CVD	114 (1.1)	19 (1.2)	40 (1.3)	55 (1.0)	0.440	83 (1.0)	31 (2.0)	0.002

Data are presented as *n* (column %) unless otherwise indicated

^a^Baseline characteristics for all population

^b^Inactive, 0 h/week of MVPA; below recommendations, 1–2 h/week of MVPA and <2 h/week of VPA; following recommendations, ≥3 h/week of MVPA or ≥2 h/week of VPA

As there was no evidence of an interaction of physical activity measures with sex or age (*p* for interaction >0.21), analyses were undertaken on the total study population. The Schoenfeld’s test suggested that the proportional hazards assumption was not violated (*p* = 0.07–0.78). The shape of the associations of MPA, VPA and MVPA in 1985–1988 with the incidence of diabetes is shown in ESM Figs 2–4. Overall, lower risk of diabetes was observed starting at 1 h/week of MPA, VPA or MVPA; there was a marginal further risk reduction at longer durations.

Table 2 presents results from an illness–death model on the association of MPA and VPA with risk of diabetes onset. Compared with no MPA, the risk of diabetes was up to 19% lower for MPA durations of 1 h or more per week (HRs ranging from 0.81 to 0.96) in analysis adjusted for sociodemographic, behavioural and VPA variables. Further adjustment for BMI slightly reduced the risk estimates, which were no longer statistically significant. No further changes were observed on adjustment for cardiovascular health variables.

**Table 2:** Association of MPA and VPA with incident type 2 diabetes^a^ (*N* = 9987)

Activity level	Diabetes incidence (*n* cases/N total)	Unadjusted		Adjusted for sociodemographic and behavioural variables^b^		Additionally adjusted for BMI		Fully adjusted^c^	
		HR (95% CI)	*p* value	HR (95% CI)	*p* value	HR (95% CI)	*p* value	HR (95% CI)	*p* value
MPA									
0 h/week	381/1894	1.00		1.00		1.00		1.00	
1 h/week	286/2112	0.54 (0.46, 0.63)*	<0.001	0.81 (0.69, 0.96)*	0.012	0.86 (0.73, 1.01)	0.070	0.87 (0.73, 1.03)	0.110
2 h/week	340/2145	0.65 (0.56, 0.75)*	<0.001	0.96 (0.83, 1.13)	0.641	1.01 (0.85, 1.18)	0.949	1.01 (0.84, 1.23)	0.928
3–4 h/week	283/1988	0.58 (0.49, 0.67)*	<0.001	0.84 (0.72, 0.99)*	0.038	0.86 (0.73, 1.01)	0.071	0.86 (0.72, 1.02)	0.089
≥5 h/week	263/1848	0.57 (0.49, 0.67)*	<0.001	0.82 (0.69, 0.97)*	0.021	0.84 (0.71, 1.00)	0.051	0.84 (0.70, 1.00)	0.054
VPA									
0 h/week	981/5688	1.00		1.00		1.00		1.00	
1 h/week	302/2207	0.66 (0.58, 0.75)*	<0.001	0.87 (0.76, 1.00)*	0.048	0.92 (0.80, 1.05)	0.215	0.93 (0.81, 1.07)	0.294
≥2 h/week	270/2092	0.63 (0.55, 0.72)*	<0.001	0.81 (0.70, 0.93)*	0.004	0.85 (0.73, 0.98)*	0.022	0.86 (0.74, 0.99)*	0.037
Physically inactive vs active^d^									
Inactive (no MVPA)	339/1607	1.00		1.00		1.00		1.00	
Active (any MVPA)	1214/8380	0.54 (0.48, 0.61)*	<0.001	0.80 (0.70, 0.91)*	0.001	0.85 (0.74, 0.96)*	0.012	0.85 (0.75, 0.97)*	0.018
Following physical activity recommendations^e^									
No	777/4613	1.00		1.00		1.00		1.00	
Yes	776/5374	0.77 (0.70, 0.85)*	<0.001	0.90 (0.81, 1.00)*	0.049	0.90 (0.82, 1.00)	0.059	0.91 (0.82, 1.01)	0.062

Data are presented as HR (95% CI) for mean follow-up of 27.1 (SD 6.3) years

MPA, moderate intensity physical activity; VPA, vigorous intensity activity

^a^Using interval-censored, illness–death model

^b^Adjusted for age (as time-scale), sex, ethnicity, marital status, education, occupational position, smoking status, alcohol consumption, fruits and vegetables intake and mutual adjustment for MPA and VPA in analysis on these variables

^c^Additionally adjusted for hypertension, total cholesterol, CVD drugs and prevalent CVD

^d^Inactive, 0 h/week of MVPA; active, >0 h/week of MVPA

^e^No, <3 h/week of MVPA and <2 h/week of VPA; yes, ≥3 h/week of MVPA or ≥2 h/week of VPA

*p < 0.05 (all exact *p* values presented to account for multiple testing)

Compared with no VPA, practising 1 h (HR 0.87 [95% CI 0.76, 1.00]) and ≥2 h (HR 0.81 [95% CI 0.70, 0.93]) of VPA per week was associated with lower risk of diabetes in analysis adjusted for sociodemographic, behavioural and MPA measures. The association remained significant only for those practising ≥2 h/week of VPA in the fully adjusted model (HR 0.86 [95% CI 0.74, 0.99]).

Compared with no MVPA (inactive), any duration of MVPA per week (active) was associated with lower risk of diabetes in the fully adjusted model (HR 0.85 [95% CI 0.75, 0.97]). Meeting physical activity recommendations was associated with lower risk of diabetes (adjusted for sociodemographic and behavioural variables, HR 0.90 [95% CI 0.81, 1.00]), compared with not meeting these recommendations. This association was not significant after further adjustment for BMI. Sensitivity analysis based on imputed data (ESM Table 1) to account for missing physical activity and covariate data show similar findings as in the main analysis. In ESM Table 2, we report results based on Cox regression rather than an illness–death model and found some evidence of underestimation of associations in the Cox regression.

### Role of post-diabetes MVPA in subsequent risk of all-cause and CVD mortality

Of the 1553 participants who developed type 2 diabetes, 1026 had physical activity measurements available after diabetes diagnosis. In this group, those who met physical activity recommendations were more likely to have a favourable sociodemographic and health profile (ESM Table 3). Over a mean follow-up of 8.8 (SD 6.1) years, there were 165 deaths, of which 55 were due to CVD.

The proportional hazard assumption was met for all-cause mortality (Schoenfeld’s *p* values = 0.13–0.88) and although for CVD mortality the *p* value from the Schoenfeld’s test was 0.0089 for the ‘following physical activity recommendations’ category, examination of Kaplan–Meier curve did not suggest strong evidence of non-proportionality of risk. The shape of the associations of physical activity measures post-diabetes diagnosis with subsequent risk of all-cause and CVD mortality are shown in ESM Figs 5–10. Overall, cubic spline regression suggested risk reduction to start at around 1 h/week of MPA, VPA or MVPA, with the lowest risk observed at approximately 2–3 h/week and a plateauing of associations at higher durations.

Participants engaging in any amount of MVPA per week showed lower risk of all-cause mortality in fully adjusted analysis (HR 0.61 [95% CI 0.41, 0.93]) (Table 3). Lower risk of CVD mortality was observed for those following physical activity recommendations (HR 0.45 [95% CI 0.23, 0.89]) compared with those not meeting these recommendations.

**Table 3:** Association of MVPA^a^ after diabetes diagnosis with all-cause and CVD mortality (*N* = 1026)

Outcome/activity	*n* cases/*N* total	Unadjusted		Adjusted for sociodemographic and behavioural variables^b^		Additionally adjusted for BMI		Fully adjusted^c^	
		HR (95% CI)	*p* value	HR (95% CI)	*p* value	HR (95% CI)	*p* value	HR (95% CI)	*p* value
Outcome: All-cause mortality									
MVPA category^d^									
Inactive (no MVPA)	40/177	1.00		1.00		1.00		1.00	
Below recommendations	70/403	0.69 (0.47, 1.02)	0.060	0.66 (0.44, 0.99)*	0.046	0.64 (0.42, 0.98)*	0.038	0.66 (0.43, 1.01)	0.057
Following recommendations	55/446	0.48 (0.32, 0.72)*	<0.001	0.51 (0.32, 0.82)*	0.005	0.51 (0.32, 0.82)*	0.005	0.54 (0.33, 0.88)*	0.012
Physically inactive vs active^e^									
Inactive (no MVPA)	40/177	1.00		1.00		1.00		1.00	
Active (any MVPA)	125/849	0.58 (0.41, 0.83)*	0.003	0.60 (0.40, 0.88)*	0.010	0.59 (0.39, 0.88)*	0.009	0.61 (0.41, 0.93)*	0.020
Following physical activity recommendations^f^									
No	110/580	1.00		1.00		1.00		1.00	
Yes	55/446	0.62 (0.45, 0.85)*	0.003	0.70 (0.49, 0.98)*	0.040	0.70 (0.50, 1.00)	0.048	0.73 (0.51, 1.05)	0.088
Outcome: CVD mortality									
MVPA category^d^									
Inactive (no MVPA)	15/177	1.00		1.00		1.00		1.00	
Below recommendations	28/403	0.74 (0.39, 1.40)	0.355	0.81 (0.42, 1.56)	0.526	0.82 (0.42, 1.59)	0.554	0.84 (0.41, 1.70)	0.629
Following recommendations	12/446	0.29 (0.13, 0.63)*	0.002	0.36 (0.15, 0.85)*	0.019	0.36 (0.15, 0.86)*	0.021	0.40 (0.16, 0.96)*	0.041
Physically inactive vs active^e^									
Inactive (no MVPA)	15/177	1.00		1.00		1.00		1.00	
Active (any MVPA)	40/849	0.50 (0.28, 0.92)*	0.025	0.60 (0.31, 1.16)	0.132	0.61 (0.31, 1.19)	0.149	0.65 (0.32, 1.32)	0.237
Following physical activity recommendations^f^									
No	43/580	1.00		1.00		1.00		1.00	
Yes	12/446	0.35 (0.19, 0.67)*	0.001	0.41 (0.21, 0.83)*	0.013	0.41 (0.21, 0.83)*	0.012	0.45 (0.23, 0.89)*	0.022

Data are presented as HR (95% CI), estimated using inverse probabilities weighted Cox regression models, for mean follow-up of 8.8 (SD 6.1) years

^a^MVPA was taken at the first wave with available measurement after diabetes diagnosis

^b^Adjusted for age (as time-scale), sex, ethnicity, marital status, education, occupational position, smoking status, alcohol consumption, and fruits and vegetables intake

^c^Additionally adjusted for hypertension, total cholesterol, SF-36 mental and physical component summary scores, CVD drugs and prevalent CVD

^d^Inactive, 0 h/week of MVPA; below recommendations, 0.1–2.4 h/week of MVPA and <1.25 h/week of VPA; following recommendations, ≥2.5 h/week of MVPA or ≥1.25 h/week of VPA

^e^Inactive, 0 h/week of MVPA; active, >0 h/week of MVPA

^f^No, <2.5 h/week of MVPA and <1.25 h/week of VPA; yes ≥2.5 h/week of MVPA or ≥1.25 h/week of VPA

*p < 0.05 (all exact *p* values presented to account for multiple testing)

In sensitivity analyses, restricting the analysis to no more than 6 years between diabetes diagnosis and subsequent MVPA assessment yielded similar findings (ESM Table 4) to those presented in the main analysis. Repeating the analysis after excluding data for individuals with prevalent CVD (*n* = 947) showed similar findings to the main analysis of CVD mortality, although the estimates were less precise and the strength of association was attenuated (ESM Table 5). Use of multiple imputation (ESM Table 6) to account for missing data instead of IPW produced findings similar to the main analysis (Table 3).

## Discussion

This prospective study presents two key findings on the role of physical activity in the aetiology and prognosis of type 2 diabetes. First, compared with not undertaking any MVPA, engaging in even some amount of MVPA was associated with lower risk of incident type 2 diabetes over a 27 year mean follow-up. Second, among participants who developed type 2 diabetes during the follow-up, MVPA of any duration was associated with lower risk of mortality, although CVD mortality risk reduction required MVPA at recommended durations.

Several studies have examined the shape of the association between physical activity and diabetes. Meta-analysis of observational studies usually combine intensity (light, moderate, vigorous) and duration of physical activity to estimate overall level of physical activity, expressed as MET-hour/week. A meta-analysis of 28 studies (*N* = 1,261,991 individuals) suggested diabetes risk reduction starting at 2.25 MET-h/week (RR 0.93), with further risk reduction at the current recommendations (11.25 MET-h/week; RR 0.74) extending to activity greater than the recommended level (22.5 MET-h/week; RR 0.64) [7]. Another meta-analysis on 261,618 participants, which adjusted for a wider range of covariates, reported the largest difference in diabetes risk between inactive participants (0 MET-h/week) and those undertaking 6 MET-h/week of physical activity (RR 0.77), with little further benefit at the current physical activity guidelines (11.25 MET-h/week; RR 0.74) [9]. A further meta-analysis found lower risk of type 2 diabetes starting at 10 MET-h/week, with additional risk reductions observed beyond this level [8].

Few studies focused specifically on MVPA and incident diabetes [10–12]. One such study, based on 99,316 middle-aged and older women followed for 8 years, found a non-linear association between aerobic activities of moderate-to-vigorous intensity and risk of diabetes; the greatest benefit was seen in those undertaking at least some MVPA compared with none [11]. We also found MVPA of any duration to be associated with lower incidence of diabetes, with the follow-up extending to 27 years. The shape of the association for MPA and VPA, considered separately, with diabetes incidence was similar. These associations were attenuated after adjustment for BMI, suggesting that body weight plays a role in the physical activity–diabetes association. These results hint that choice of covariates in previous studies might explain some of the inconsistencies in findings on MVPA duration. Further research using objectively assessed physical activity would complement the evidence which is currently primarily based on self-reported physical activity. Large studies have started using objective measures of physical activity but these studies have follow-up periods of limited duration.

Several studies have examined the role of physical activity in the prognosis of type 2 diabetes. Most studies are on all-cause [15–18] and CVD mortality [16, 17, 19], and found physical activity to reduce the risk of these outcomes. However, the duration of physical activity that confers protection in this group remains unclear. Some studies suggest the association with all-cause [15–17] and CVD mortality [16, 17] to be linear, while other studies report a plateauing of the association [18], as observed in the present study, and still other studies found risk of all-cause mortality to be reduced to a greater extent at longer durations of physical activity [42].

Potential reasons for inconsistencies in the shape of the associations between physical activity, type 2 diabetes incidence and subsequent mortality risk include small sample sizes, misclassification of physical activity durations and intensities, and heterogeneity in the assessment of physical activity. In addition, the dose–response assessments in meta-analysis are problematic as they combine measures of physical activity that are heterogeneous (based on duration or frequency in physical activity of specific or non-specific intensity) and estimates from categorical variables are constrained to the mean or median MET or duration of the category [9], a procedure that is particularly vulnerable to bias in effect estimates for the uppermost category.

The present study has several strengths. To the best of our knowledge, the analysis on the association between physical activity and type 2 diabetes onset has the longest follow-up (mean 27.1 years). This study is based on both men and women as compared with notable previous studies with follow-up longer than 15 years based on men [43–46] or women [47] only. Ascertainment of diabetes was undertaken using multiple, objective sources that included clinical examination rather than self-reported data. The association between physical activity measures and risk of diabetes was estimated using interval-censored illness–death models to account for death as a competing risk and the interval-censored nature of the data, an approach not used in previous studies. Finally, we were able to control for selection bias in our second aim (investigating the association of MVPA post-diabetes diagnosis with subsequent mortality risk), an approach that has not been used in previous studies [15–19].

Our findings need to be interpreted in light of some limitations: physical activity was self-reported and thus subject to misclassification, and the duration of physical activity was assessed using whole numbers of hours in 1985–1988 which could have led to imprecise categorisation of duration. Small numbers do not allow firm conclusions to be drawn on CVD mortality in individuals with diabetes. Finally, as with all observational studies, bias due to residual confounding cannot be eliminated fully despite adjustment for a large range of covariates.

In conclusion, our results on the natural course of diabetes in a large sample free of diabetes at recruitment, followed for over 27 years, suggest that MVPA of any duration is an important target to prevent risk of type 2 diabetes. Among participants with type 2 diabetes, benefits of physical activity were also evident for all-cause mortality at lower durations of MVPA than those recommended, although the recommended durations were required for lowering risk of CVD mortality.

## Electronic supplementary material

ESM (PDF 876 kb)

## Data Availability

Whitehall II data, protocols and other metadata are available to the scientific community. Please refer to the Whitehall II data sharing policy at https://www.ucl.ac.uk/epidemiology-health-care/research/epidemiology-and-public-health/research/whitehall-ii/data-sharing.

## Abbreviations

CVD: Cardiovascular disease
HES: Hospital Episode Statistics
IPW: Inverse probability weighting
MCS: Mental component summary
MET: Metabolic equivalent
MPA: Moderate physical activity
MVPA: Moderate-to-vigorous physical activity
NHS: National Health Service
PCS: Physical component summary
SF-36: 36-Item Short Form Health Survey
VPA: Vigorous physical activity

## References

[1] International Diabetes Federation (2017). IDF diabetes atlas.

[2] Emerging Risk Factors Collaboration (2010). Diabetes mellitus, fasting blood glucose concentration, and risk of vascular disease: a collaborative meta-analysis of 102 prospective studies. Lancet.

[3] International Diabetes Federation (2016). Diabetes and cardiovascular disease.

[4] American Diabetes Association (2019) 5. Lifestyle Management: Standards of Medical Care in Diabetes—2019. Diabetes Care 42(Supplement 1):S46–S60. 10.2337/dc19-S005 10.2337/dc19-S005 30559231

[5] Zheng Y, Ley SH, Hu FB (2018). Global aetiology and epidemiology of type 2 diabetes mellitus and its complications. Nat Rev Endocrinol.

[6] Colberg SR, Sigal RJ, Yardley JE (2016). Physical activity/exercise and diabetes: a position statement of the American Diabetes Association. Diabetes Care.

[7] Smith AD, Crippa A, Woodcock J, Brage S (2016). Physical activity and incident type 2 diabetes mellitus: a systematic review and dose-response meta-analysis of prospective cohort studies. Diabetologia.

[8] Kyu HH, Bachman VF, Alexander LT (2016). Physical activity and risk of breast cancer, colon cancer, diabetes, ischemic heart disease, and ischemic stroke events: systematic review and dose-response meta-analysis for the Global Burden of Disease Study 2013. BMJ.

[9] Wahid A, Manek N, Nichols M et al (2016) Quantifying the association between physical activity and cardiovascular disease and diabetes: a systematic review and meta-analysis. J Am Heart Assoc 5(9). 10.1161/jaha.115.002495 10.1161/JAHA.115.002495 PMC5079002 27628572

[10] Ding D, Chong S, Jalaludin B, Comino E, Bauman AE (2015) Risk factors of incident type 2-diabetes mellitus over a 3-year follow-up: Results from a large Australian sample. Diabetes Res Clin Pract 108(2):306–315. 10.1016/j.diabres.2015.02.002 10.1016/j.diabres.2015.02.002 25737033

[11] Grøntved A, Pan A, Mekary RA (2014). Muscle-strengthening and conditioning activities and risk of type 2 diabetes: a prospective study in two cohorts of US women. PLoS Med.

[12] Demakakos P, Hamer M, Stamatakis E, Steptoe A (2010). Low-intensity physical activity is associated with reduced risk of incident type 2 diabetes in older adults: evidence from the English Longitudinal Study of Ageing. Diabetologia.

[13] Leffondré K, Touraine C, Helmer C, Joly P (2013). Interval-censored time-to-event and competing risk with death: is the illness-death model more accurate than the Cox model?. Int J Epidemiol.

[14] Gill JM, Cooper AR (2008). Physical activity and prevention of type 2 diabetes mellitus. Sports Med.

[15] Sone H, Tanaka S, Suzuki S (2013). Leisure-time physical activity is a significant predictor of stroke and total mortality in Japanese patients with type 2 diabetes: analysis from the Japan Diabetes Complications Study (JDCS). Diabetologia.

[16] Sadarangani KP, Hamer M, Mindell JS, Coombs NA, Stamatakis E (2014). Physical activity and risk of all-cause and cardiovascular disease mortality in diabetic adults from Great Britain: pooled analysis of 10 population-based cohorts. Diabetes Care.

[17] Hu G, Jousilahti P, Barengo NC, Qiao Q, Lakka TA, Tuomilehto J (2005). Physical activity, cardiovascular risk factors, and mortality among Finnish adults with diabetes. Diabetes Care.

[18] Tanasescu M, Leitzmann MF, Rimm EB, Hu FB (2003). Physical activity in relation to cardiovascular disease and total mortality among men with type 2 diabetes. Circulation.

[19] Liu G, Li Y, Hu Y et al (2018) Influence of lifestyle on incident cardiovascular disease and mortality in patients with diabetes mellitus. J Am Coll Cardiol 71(25):2867–2876. 10.1016/j.jacc.2018.04.027 10.1016/j.jacc.2018.04.027 PMC6052788 29929608

[20] Tuomilehto J, Lindström J, Eriksson JG (2001). Prevention of type 2 diabetes mellitus by changes in lifestyle among subjects with impaired glucose tolerance. N Engl J Med.

[21] Li G, Zhang P, Wang J (2014). Cardiovascular mortality, all-cause mortality, and diabetes incidence after lifestyle intervention for people with impaired glucose tolerance in the Da Qing Diabetes Prevention Study: a 23-year follow-up study. Lancet Diabetes Endocrinol.

[22] Balk EM, Earley A, Raman G, Avendano EA, Pittas AG, Remington PL (2015). Combined diet and physical activity promotion programs to prevent type 2 diabetes among persons at increased risk: a systematic review for the Community Preventive Services Task Force. Ann Intern Med.

[23] Gæde P, Lund-Andersen H, Parving H-H, Pedersen O (2008). Effect of a multifactorial intervention on mortality in type 2 diabetes. N Engl J Med.

[24] Schellenberg ES, Dryden DM, Vandermeer B, Ha C, Korownyk C (2013). Lifestyle interventions for patients with and at risk for type 2 diabetes: a systematic review and meta-analysis. Ann Intern Med.

[25] Marmot M, Brunner E (2005). Cohort profile: the Whitehall II study. Int J Epidemiol.

[26] Bouillon K, Singh-Manoux A, Jokela M (2011). Decline in low-density lipoprotein cholesterol concentration: lipid-lowering drugs, diet, or physical activity? Evidence from the Whitehall II study. Heart.

[27] Hamer M, Brunner EJ, Bell J et al (2013) Physical activity patterns over 10 years in relation to body mass index and waist circumference: the Whitehall II cohort study. Obesity 21(12):E755–E761. 10.1002/oby.20446 10.1002/oby.20446 23512753

[28] Hamer M, Sabia S, Batty GD (2012). Physical activity and inflammatory markers over 10 years: follow-up in men and women from the Whitehall II cohort study. Circulation.

[29] Rennie KL, Hemingway H, Kumari M, Brunner E, Malik M, Marmot M (2003). Effects of moderate and vigorous physical activity on heart rate variability in a British study of civil servants. Am J Epidemiol.

[30] Richardson MT, Leon AS, Jacobs DR, Ainsworth BE, Serfass R (1994). Comprehensive evaluation of the Minnesota leisure time physical activity questionnaire. J Clin Epidemiol.

[31] Ainsworth BE, Haskell WL, Whitt MC (2000). Compendium of physical activities: an update of activity codes and MET intensities. Med Sci Sports Exerc.

[32] Shiroma EJ, Cook NR, Manson JE, Buring JE, Rimm EB, Lee I-M (2015) Comparison of self-reported and accelerometer-assessed physical activity in older women. PLoS One 10(12):e0145950. 10.1371/journal.pone.0145950 10.1371/journal.pone.0145950 PMC4694656 26713857

[33] Pate RR, Pratt M, Blair SN et al (1995) Physical activity and public health: a recommendation from the Centers for Disease Control and Prevention and the American College of Sports Medicine. JAMA 273(5):402–407. 10.1001/jama.273.5.402 10.1001/jama.273.5.402 7823386

[34] World Health Organization (2010). Global recommendations on physical activity for health.

[35] World Health Organization (2006). Definition and diagnosis of diabetes mellitus and intermediate hyperglycaemia: report of a WHO/IDF consultation.

[36] Williams ED, Rawal L, Oldenburg BF, Renwick C, Shaw JE, Tapp RJ (2012). Risk of Cardiovascular and All-Cause Mortality: Impact of Impaired Health-Related Functioning and Diabetes Mellitus: The Australian Diabetes Obesity and Lifestyle Study (AUSDIAB). Diabetes Care.

[37] Harrell FE (2001). Restricted cubic splines. Regression modeling strategies.

[38] Touraine C, Gerds TA, Joly P (2013). The SmoothHazard package for R: fitting regression models to interval-censored observations of illness-death models.

[39] Boucquemont J, Metzger M, Combe C, Stengel B, Leffondre K, NephroTest Study Group (2014). Should we use standard survival models or the illness-death model for interval-censored data to investigate risk factors of chronic kidney disease progression?. PLoS One.

[40] Seaman SR, White IR (2013). Review of inverse probability weighting for dealing with missing data. Stat Methods Med Res.

[41] Williams ED, Rawal L, Oldenburg BF, Renwick C, Shaw JE, Tapp RJ (2012). Risk of cardiovascular and all-cause mortality: impact of impaired health-related functioning and diabetes: the Australian Diabetes, Obesity and Lifestyle (AusDiab) study. Diabetes Care.

[42] Kodama S, Tanaka S, Heianza Y (2013). Association between physical activity and risk of all-cause mortality and cardiovascular disease in patients with diabetes: a meta-analysis. Diabetes Care.

[43] Siegel LC, Sesso HD, Bowman TS, Lee I-M, Manson JE, Gaziano JM (2009). Physical activity, body mass index, and diabetes risk in men: a prospective study. Am J Med.

[44] Lynch J, Helmrich SP, Lakka TA (1996). Moderately intense physical activities and high levels of cardiorespiratory fitness reduce the risk of non-insulin-dependent diabetes mellitus in middle-aged men. Arch Intern Med.

[45] Wannamethee SG, Shaper AG, Alberti KG (2000). Physical activity, metabolic factors, and the incidence of coronary heart disease and type 2 diabetes. Arch Intern Med.

[46] Grøntved A, Rimm EB, Willett WC, Andersen LB, Hu FB (2012). A prospective study of weight training and risk of type 2 diabetes mellitus in men. Arch Intern Med.

[47] Folsom AR, Kushi LH, Hong CP (2000). Physical activity and incident diabetes mellitus in postmenopausal women. Am J Public Health.

