# Supplementary material for: Association of moderate and vigorous physical activity with incidence of type 2 diabetes and subsequent mortality: 27 year follow-up of the Whitehall II study
Source: Diabetologia. 2019 Dec 2;63(3):537–48. doi: 10.1007/s00125-019-05050-1 (PMC6997261; doi:10.1007/s00125-019-05050-1)
Supplement: Supplementary file 1 — (PDF 876 kb) [file 125_2019_5050_MOESM1_ESM.pdf]

ESM Table 1: Association of MPA and VPA with incident type 2 diabetes[a] using imputed data (N=10208)

Activity Level	N cases/ N total	Unadjusted		Adjusted for sociodemographic and behavioural variables[b]		Additionally adjusted for BMI		Fully adjusted[c]	
		HR (95% CI)	*p* value	HR (95% CI)	*p* value	HR (95% CI)	*p* value	HR (95% CI)	*p* value
**MPA**									
0 hour/week	394/1963	1.00 (ref)		1.00 (ref)		1.00 (ref)		1.00 (ref)	
1 hour/week	291/2133	0.54 (0.46-0.63)*	<0.001	0.82 (0.70-0.96)*	0.013	0.86 (0.74-1.00)*	0.048	0.87 (0.74-1.02)	0.081
2 hours/week	347/2177	0.65 (0.56-0.75)*	<0.001	0.96 (0.83-1.12)	0.625	1.01 (0.90-1.12)	0.904	1.01 (0.87-1.17)	0.902
3-4 hours/week	291/2029	0.58 (0.50-0.68)*	<0.001	0.85 (0.73-1.00)*	0.047	0.87 (0.74-1.01)	0.062	0.87 (0.74-1.01)	0.075
≥5 hours/week	270/1906	0.57 (0.49-0.67)*	<0.001	0.82 (0.69-0.96)*	0.016	0.84 (0.72-0.98)*	0.028	0.83 (0.71-0.98)*	0.031
**VPA**									
0 hour/week	999/5787	1.00 (ref)		1.00 (ref)		1.00 (ref)		1.00 (ref)	
1 hour/week	311/2252	0.67 (0.59-0.76)*	<0.001	0.87 (0.76-1.00)*	0.045	0.92 (0.80-1.05)	0.207	0.93 (0.81-1.06)	0.273
≥2 hours/week	283/2169	0.64 (0.56-0.73)*	<0.001	0.81 (0.70-0.93)*	0.003	0.85 (0.74-0.97)*	0.019	0.86 (0.75-0.98)*	0.029
**Physically inactive vs active[d]**									
Inactive (No MVPA)	345/1644	1.00 (ref)		1.00 (ref)		1.00 (ref)		1.00 (ref)	
Active (Any MVPA)	1248/8564	0.55 (0.48-0.61)*	<0.001	0.80 (0.70-0.91)*	0.001	0.85 (0.75-0.97)*	0.013	0.85 (0.75-0.97)*	0.017
**Following physical activity recommendations[e]**									
No	794/4690	1.00 (ref)		1.00 (ref)		1.00 (ref)		1.00 (ref)	
Yes	799/5518	0.77 (0.70-0.85)*	<0.001	0.89 (0.81-0.99)*	0.030	0.90 (0.81-1.00)*	0.043	0.90 (0.81-1.00)*	0.045

Data are presented as HR (95% CI) for mean follow-up of 26.8 (SD 6.3) years

MPA, moderate intensity physical activity; VPA, vigorous intensity activity

[a]Using interval-censored, illness-deathmodel

[b]Adjusted for age (as time-scale), sex, ethnicity, marital status, education, occupational position, smoking status, alcohol consumption, fruits and vegetables intake and mutual adjustment for MPA and VPA in analysis on these variables

[c]Additionally adjusted for hypertension, total cholesterol, CVD drugs and prevalent CVD

[d]Inactive "0 hour/week of MVPA"; Active ">0 hours/week of MVPA"

[e]No "<3 hours/week of MVPA and <2 hours/week of VPA"; Yes "≥3 hours/week of MVPA or ≥2 hours/week of VPA"

*p< 0.05, all exact p values presented to take into account multiple testing

ESM Table 2: Association of MPA and VPA with incident type 2 diabetes using Cox regression[a] (N=9987)

Activity Level	N cases/ N total	Unadjusted		Adjusted for sociodemographic and behavioural variables[b]		Additionally adjusted for BMI		Fully adjusted[c]	
		**HR (95% CI)**	***p* value**	**HR (95% CI)**	***p* value**	**HR (95% CI)**	***p* value**	**HR (95% CI)**	***p* value**
**MPA**									
0 hour/week	381/1894	1.00 (ref)		1.00 (ref)		1.00 (ref)		1.00 (ref)	
1 hour/week	286/2112	0.62 (0.54-0.73)*	<0.001	0.83 (0.71-0.98)*	0.025	0.88 (0.75-1.03)	0.112	0.88 (0.75-1.03)	0.113
2 hours/week	340/2145	0.74 (0.64-0.86)*	<0.001	0.97 (0.83-1.14)	0.732	1.02 (0.87-1.19)	0.833	1.01 (0.87-1.18)	0.880
3-4 hours/week	283/1988	0.66 (0.57-0.77)*	<0.001	0.85 (0.73-1.01)	0.060	0.88 (0.75-1.04)	0.133	0.88 (0.75-1.03)	0.120
≥5 hours/week	263/1848	0.66 (0.57-0.78)*	<0.001	0.87 (0.73-1.03)	0.093	0.90 (0.76-1.07)	0.222	0.89 (0.75-1.05)	0.173
**VPA**									
0 hour/week	981/5688	1.00 (ref)		1.00 (ref)		1.00 (ref)		1.00 (ref)	
1 hour/week	302/2207	0.76 (0.66-0.86)*	<0.001	0.91 (0.80-1.05)	0.191	0.95 (0.83-1.09)	0.503	0.96 (0.84-1.11)	0.597
≥2 hours/week	270/2092	0.71 (0.62-0.81)*	<0.001	0.84 (0.73-0.96)*	0.014	0.88 (0.76-1.01)	0.068	0.89 (0.77-1.03)	0.109
**Physically inactive vs active[d]**									
Inactive (No MVPA)	339/1607	1.00 (ref)		1.00 (ref)		1.00 (ref)		1.00 (ref)	
Active (Any MVPA)	1214/8380	0.63 (0.56-0.71)*	<0.001	0.83 (0.73-0.94)*	0.004	0.88 (0.77-1.00)	0.058	0.88 (0.77-1.00)	0.058
**Following physical activity recommendations[e]**									
No	777/4613	1.00 (ref)		1.00 (ref)		1.00 (ref)		1.00 (ref)	
Yes	776/5374	0.83 (0.75-0.91)*	<0.001	0.92 (0.83-1.02)	0.121	0.94 (0.85-1.04)	0.233	0.94 (0.85-1.04)	0.245

Data are presented as HR (95% CI) for mean follow0up of 27.1 (SD 6.3) years
MPA, moderate intensity physical activity; VPA, vigorous intensity activity
[a]Instead of interval-censored, illness-death model
[b]Adjusted for age (as time-scale), sex, ethnicity, marital status, education, occupational position, smoking status, alcohol consumption, fruits & vegetables intake and mutual adjustment for MPA and VPA in analysis on these variables
[c]Additionally adjusted for hypertension, total cholesterol, CVD drugs and prevalent CVD
[d]Inactive “0 hour/week of MVPA”; Active “>0 hours/week of MVPA”
[e]No “<3 hours/week of MVPA and <2 hours/week of VPA”; Yes “≥3 hours/week of MVPA or ≥2 hours/week of VPA”
*p< 0.05, all exact p values presented to take into account multiple testing

ESM Table 3: Sample characteristics for the analysis of moderate-to-vigorous physical activity post-diabetes diagnosis with subsequent mortality (N=1026)

Characteristics	Total population	Moderate-to-vigorous physical activity (MVPA)[a]			
		**Inactive (No MVPA)**	**Below recommendations**	**Following recommendations**	***p* value**
**N (row %)**	1026	177 (17.3)	403 (39.3)	446 (43.5)	
Age (years), M(SD)	65.7 (8.1)	66.0 (9.4)	65.6 (7.7)	65.8 (7.9)	0.934
Non-white	205 (20.0)	55 (31.1)	80 (19.9)	70 (15.7)	<0.001
Female	317 (30.9)	73 (41.2)	141 (35.0)	103 (23.1)	<0.001
Married/cohabitating	735 (71.6)	104 (58.8)	290 (72.0)	341 (76.5)	<0.001
University degree or higher	250 (24.4)	42 (23.7)	105 (26.1)	103 (23.1)	0.590
Low occupational position	189 (18.4)	66 (37.3)	77 (19.1)	46 (10.3)	<0.001
Current smokers	75 (7.3)	17 (9.6)	31 (7.7)	27 (6.1)	0.286
>14 units of alcohol per week	208 (20.3)	25 (14.1)	85 (21.1)	98 (22.0)	0.078
<daily fruits & vegetables intake	262 (25.5)	71 (40.1)	104 (25.8)	87 (19.5)	<0.001
BMI≥30.0 kg/m^2	353 (34.4)	74 (41.8)	150 (37.2)	129 (28.9)	0.003
Hypertension	677 (66.0)	121 (68.4)	274 (65.2)	282 (65.7)	0.755
Total cholesterol (mmol/L), M(SD)	5.0 (1.3)	5.2 (1.3)	5.1 (1.4)	4.9 (1.2)	0.015
CVD drugs	745 (72.6)	132 (74.6)	299 (71.2)	314 (73.2)	0.656
Prevalent CVD	79 (7.7)	20 (11.3)	27 (6.7)	32 (7.1)	0.138
Physical component score of SF-36, M(SD)	44.7 (10.6)	40.5 (11.9)	44.3 (10.7)	46.6 (9.3)	<0.001
Mental component score of SF-36, M(SD)	52.3 (9.2)	50.5 (10.7)	51.6 (9.5)	53.8 (7.8)	0.002

Data are presented as n (column %) unless otherwise indicated

[a]Inactive “0 hour/week of MVPA”; Below recommendations “0.1 to 2.4 hours/week of MVPA and <1.25 hours/week of VPA”; Following recommendations “≥2.5 hours/week of MVPA or ≥1.25 hours/week of VPA”

ESM Table 4: Association of MVPA[a] with all-cause and CVD mortality excluding participants with MVPA measurement assessed more than 6 years post-diabetes diagnosis (N=939)

Outcome/activity	N cases/ N total	Unadjusted		Adjusted for sociodemographic and behavioural variables[b]		Additionally adjusted for BMI		Fully adjusted[c]	
		HR (95% CI)	*p* value	HR (95% CI)	*p* value	HR (95% CI)	*p* value	HR (95% CI)	*p* value
**Outcome: All-cause mortality**									
**MVPA category[d]**									
Inactive (No MVPA)	35/157	1.00 (ref)		1.00 (ref)		1.00 (ref)		1.00 (ref)	
Below recommendations	61/370	0.70 (0.46-1.05)	0.087	0.69 (0.44-1.08)	0.102	0.68 (0.43-1.06)	0.091	0.70 (0.44-1.12)	0.138
Following recommendations	50/412	0.51 (0.33-0.79)*	0.002	0.59 (0.36-0.97)*	0.036	0.59 (0.36-0.97)*	0.039	0.62 (0.36-1.04)	0.070
**Physically inactive vs Active[e]**									
Inactive (No MVPA)	35/157	1.00 (ref)		1.00 (ref)		1.00 (ref)		1.00 (ref)	
Active (Any MVPA)	111/782	0.60 (0.41-0.88)*	0.009	0.65 (0.42-0.99)*	0.046	0.64 (0.41-0.99)*	0.045	0.67 (0.42-1.05)	0.081
**Following physical activity recommendations[f]**									
No	96/527	1.00 (ref)		1.00 (ref)		1.00 (ref)		1.00 (ref)	
Yes	50/412	0.66 (0.47-0.92)*	0.014	0.77 (0.54-1.11)	0.161	0.78 (0.54-1.13)	0.190	0.80 (0.55-1.17)	0.250
**Outcome: CVD mortality**									
**MVPA categorys[d]**									
Inactive (No MVPA)	12/157	1.00 (ref)		1.00 (ref)		1.00 (ref)		1.00 (ref)	
Below recommendations	23/370	0.81 (0.40-1.62)	0.545	0.89 (0.42-1.90)	0.772	0.93 (0.44-2.00)	0.859	1.01 (0.45-2.28)	0.979
Following recommendations	10/412	0.32 (0.14-0.76)*	0.010	0.40 (0.15-1.10)	0.076	0.41 (0.15-1.12)	0.083	0.45 (0.17-1.22)	0.118
**Physically inactive vs Active[e]**									
Inactive (No MVPA)	12/157	1.00 (ref)		1.00 (ref)		1.00 (ref)		1.00 (ref)	
Active (Any MVPA)	33/782	0.55 (0.28-1.07)	0.080	0.68 (0.31-1.46)	0.321	0.70 (0.32-1.54)	0.377	0.77 (0.34-1.76)	0.541
**Following physical activity recommendations[f]**									
No	35/527	1.00 (ref)		1.00 (ref)		1.00 (ref)		1.00 (ref)	
Yes	10/412	0.37 (0.18-0.76)*	0.006	0.44 (0.20-0.94)*	0.035	0.43 (0.20-0.92)*	0.031	0.45 (0.22-0.92)*	0.030

Data are presented as HR (95% CI) for mean follow-up of 9.0 (SD 6.1) years, estimated using inverse probabilities weighted cox regression models

[a]MVPA was taken at the first wave with available measurement after diabetes diagnosis

[b]Adjusted for age (as time-scale), sex, ethnicity, marital status, education, occupational position, smoking status, alcohol consumption, fruits & vegetables intake

[c]Additionally adjusted for hypertension, total cholesterol, SF-36 mental and physical component summary scores, CVD drugs and prevalent CVD

[d]Inactive "0 hour/week of MVPA"; Below recommendations "0.1 to 2.4 hours/week of MVPA and <1.25 hours/week of VPA"; Following recommendations "≥2.5 hours/week of MVPA or ≥1.25 hours/week of VPA"

[e]Inactive "0 hour/week of MVPA"; Active ">0 hour/week of MVPA"

[f]No "<2.5 hours/week of MVPA and <1.25 hours/week of VPA"; Yes "≥2.5 hours/week of MVPA or ≥1.25 hours/week of VPA" *p< 0.05

ESM Table 5: Association of MVPA[a] post-diabetes diagnosis with CVD mortality, excluding participants with prevalent CVD (N=947)

Outcome/activity	N cases/ N total	Unadjusted		Adjusted for sociodemographic and behavioural variables[b]		Additionally adjusted for BMI		Fully adjusted[c]	
		HR (95% CI)	*p* value	HR (95% CI)	*p* value	HR (95% CI)	*p* value	HR (95% CI)	*p* value
**Outcome: CVD mortality**									
**MVPA categories[d]**									
Inactive	12/157	1.00 (ref)		1.00 (ref)		1.00 (ref)		1.00 (ref)	
Below recommendations	23/376	0.75 (0.37-1.52)	0.430	0.84 (0.39-1.78)	0.643	0.86 (0.40-1.85)	0.698	0.96 (0.42-2.23)	0.928
Following recommendations	12/414	0.35 (0.15-0.81)*	0.014	0.45 (0.18-1.15)	0.094	0.45 (0.18-1.16)	0.100	0.53 (0.20-1.41)	0.202
**Physically inactive vs active[e]**									
Inactive (No MVPA)	12/157	1.00 (ref)		1.00 (ref)		1.00 (ref)		1.00 (ref)	
Active (Any MVPA)	35/790	0.54 (0.28-1.06)	0.074	0.66 (0.31-1.41)	0.283	0.67 (0.31-1.46)	0.318	0.78 (0.33-1.80)	0.556
**Following physical activity recommendations[f]**									
No	35/533	1.00 (ref)		1.00 (ref)		1.00 (ref)		1.00 (ref)	
Yes	12/414	0.43 (0.22-0.83)*	0.012	0.51 (0.25-1.05)	0.067	0.51 (0.25-1.03)	0.062	0.54 (0.27-1.09)	0.084

Data are presented as HR (95% CI), estimated using inverse probabilities weighted cox regression models

[a]MVPA was taken at the first wave with available measurement after diabetes diagnosis

[b]Adjusted for age (as time-scale), sex, ethnicity, marital status, education, occupational position, smoking status, alcohol consumption, fruits & vegetables intake

[c]Additionally adjusted for hypertension, total cholesterol, SF-36 mental and physical component summary scores, CVD drugs

[d]Inactive "0 hour/week of MVPA"; Below recommendations "0.1 to 2.4 hours/week of MVPA and <1.25 hours/week of VPA"; Following recommendations "≥2.5 hours/week of MVPA or ≥1.25 hours/week of VPA"

[e]Inactive "0 hour/week of MVPA"; Active ">0 hour/week of MVPA"

[f]No "<2.5 hours/week of MVPA and <1.25 hours/week of VPA"; Yes "≥2.5 hours/week of MVPA or ≥1.25 hours/week of VPA"

*p< 0.05, all exact p values presented to take into account multiple testing

ESM Table 6: Association of MVPA[a] after diabetes diagnosis with all-cause and CVD mortality (N=1553) using multiple imputed data

Outcome/activity	Unadjusted		Adjusted for sociodemographic and behavioural variables[b]		Additionally adjusted for BMI		Fully adjusted[c]	
	HR (95% CI)	*p* value	HR (95% CI)	*p* value	HR (95% CI)	*p* value	HR (95% CI)	*p* value
**Outcome: All-cause mortality**								
**MVPA categories[d]**								
Inactive (No MVPA)	1.00 (ref)		1.00 (ref)		1.00 (ref)		1.00 (ref)	
Below recommendations	0.70 (0.50-0.98)	0.037	0.71 (0.49-1.03)	0.073	0.68 (0.46-1.03)	0.066	0.71 (0.47-1.07)	0.103
Following recommendations	0.52 (0.35-0.77)*	0.001	0.55 (0.35-0.86)*	0.010	0.56 (0.36-0.86)*	0.008	0.60 (0.38-0.94)*	0.025
**Physically inactive vs Active[e]**								
Inactive (No MVPA)	1.00 (ref)		1.00 (ref)		1.00 (ref)		1.00 (ref)	
Active (Any MVPA)	0.61 (0.44-0.84)*	0.003	0.64 (0.44-0.93)*	0.020	0.63 (0.43-0.92)*	0.017	0.67 (0.45-0.98)*	0.040
**Following physical activity recommendations[f]**								
No	1.00 (ref)		1.00 (ref)		1.00 (ref)		1.00 (ref)	
Yes	0.66 (0.49-0.88)*	0.006	0.70 (0.51-0.96)*	0.028	0.74 (0.53-1.02)	0.067	0.77 (0.55-1.08)	0.130
**Outcome: CVD mortality**								
**MVPA categories[d]**								
Inactive (No MVPA)	1.00 (ref)		1.00 (ref)		1.00 (ref)		1.00 (ref)	
Below recommendations	0.70 (0.39-1.27)	0.241	0.77 (0.41-1.44)	0.416	0.83 (0.42-1.63)	0.591	0.88 (0.44-1.75)	0.707
Following recommendations	0.35 (0.17-0.70)*	0.004	0.41 (0.19-0.90)*	0.026	0.37 (0.16-0.84)*	0.018	0.41 (0.18-0.96)*	0.041
**Physically inactive vs Active[e]**								
Inactive (No MVPA)	1.00 (ref)		1.00 (ref)		1.00 (ref)		1.00 (ref)	
Active (Any MVPA)	0.52 (0.29-0.92)*	0.025	0.61 (0.33-1.13)	0.115	0.62 (0.33-1.19)	0.154	0.68 (0.35-1.34)	0.267
**Following physical activity recommendations[f]**								
No	1.00 (ref)		1.00 (ref)		1.00 (ref)		1.00 (ref)	
Yes	0.43 (0.24-0.78)*	0.005	0.49 (0.26-0.91)*	0.024	0.43 (0.22-0.82)*	0.011	0.46 (0.23-0.90)*	0.023

Data are presented as HR (95% CI), estimated using cox regression models

[a]MVPA was taken at the first wave with available measurement after diabetes diagnosis

[b]Adjusted for age (as time-scale), sex, ethnicity, marital status, education, occupational position, smoking status, alcohol consumption, fruits & vegetables intake

[c]Additionally adjusted for hypertension, total cholesterol, SF-36 mental and physical component summary scores, CVD drugs and prevalent CVD

[d]Inactive “0 hour/week of MVPA”; Below recommendations “0.1 to 2.4 hours/week of MVPA and <1.25 hours/week of VPA”; Following recommendations “≥2.5 hours/week of MVPA or ≥1.25 hours/week of VPA” [e]Inactive “0 hour/week of MVPA”; Active “>0 hour/week of MVPA”

[f]No “<2.5 hours/week of MVPA and <1.25 hours/week of VPA”; Yes “≥2.5 hours/week of MVPA or ≥1.25 hours/week of VPA”

*p< 0.05, all exact p values presented to take into account multiple testing

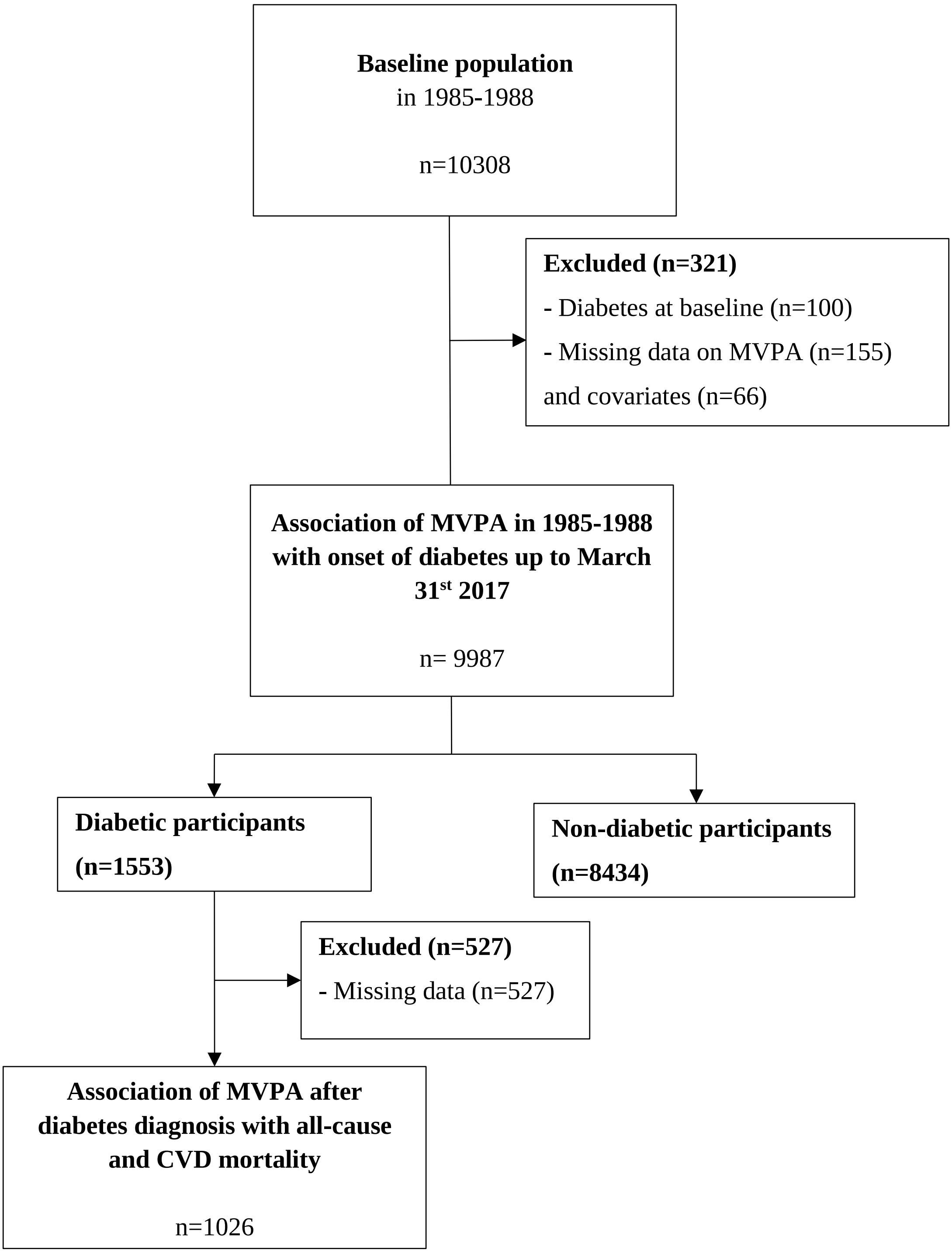

ESM Fig 1: Study flow chart of sample selection

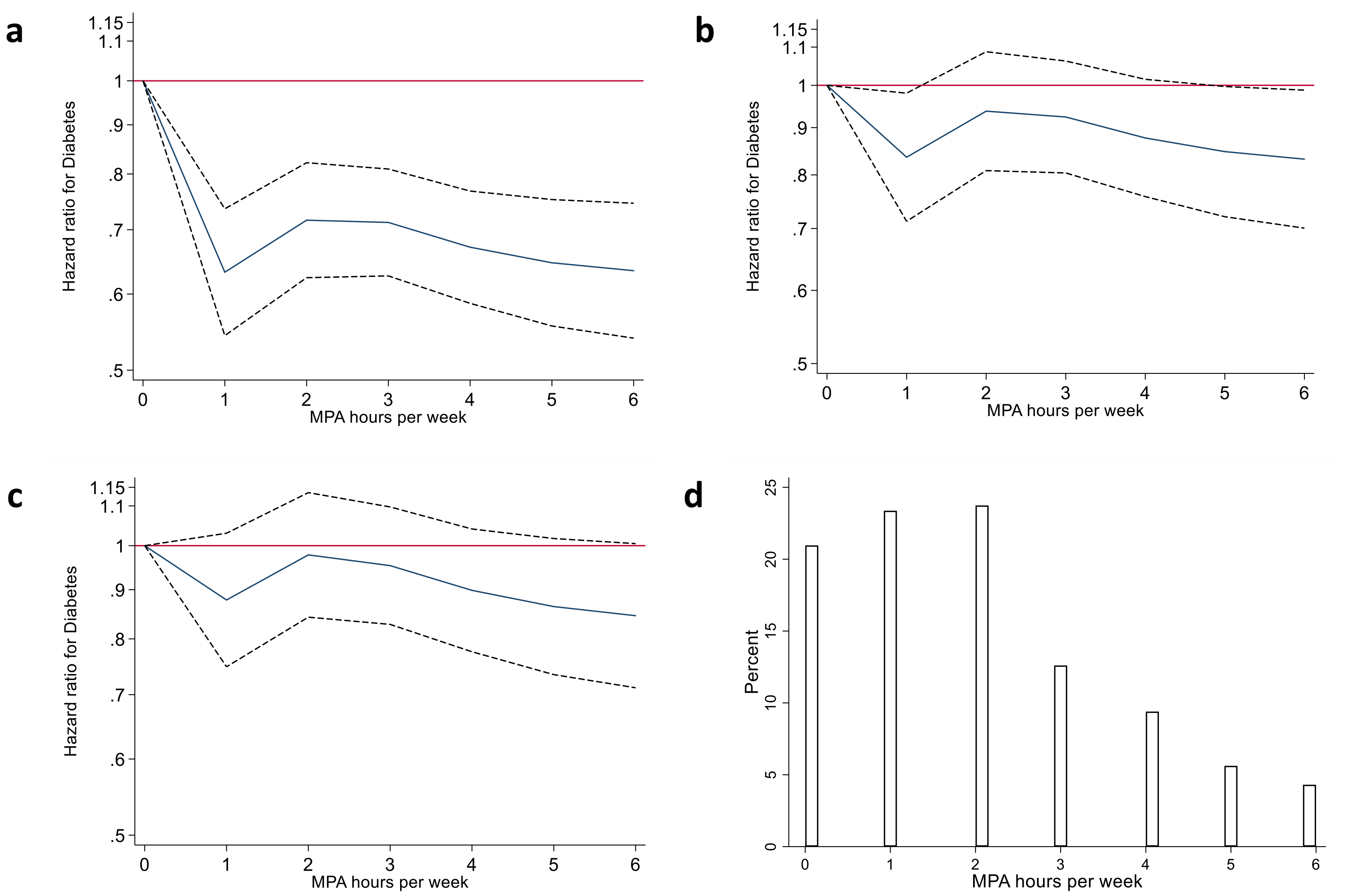

ESM Fig 2: Dose-response relation of moderate physical activity with risk of incident diabetes. (**a**) crude model; (**b**) adjusted for age (time-scale), sex, ethnicity, education, occupational grade, marital status, smoking status, alcohol consumption, fruits & vegetables intake and VPA; (**c**) additionally adjusted for BMI, hypertension, total cholesterol, CVD drugs, and prevalent CVD; (**d**) population distribution. Blue line represents the HR using restricted cubic spline regression models (5 knots). The reference value (HR=1) is 0 hour/week. Black dash lines are 95 % CI. MPA, moderate physical activity; VPA, vigorous physical activity.

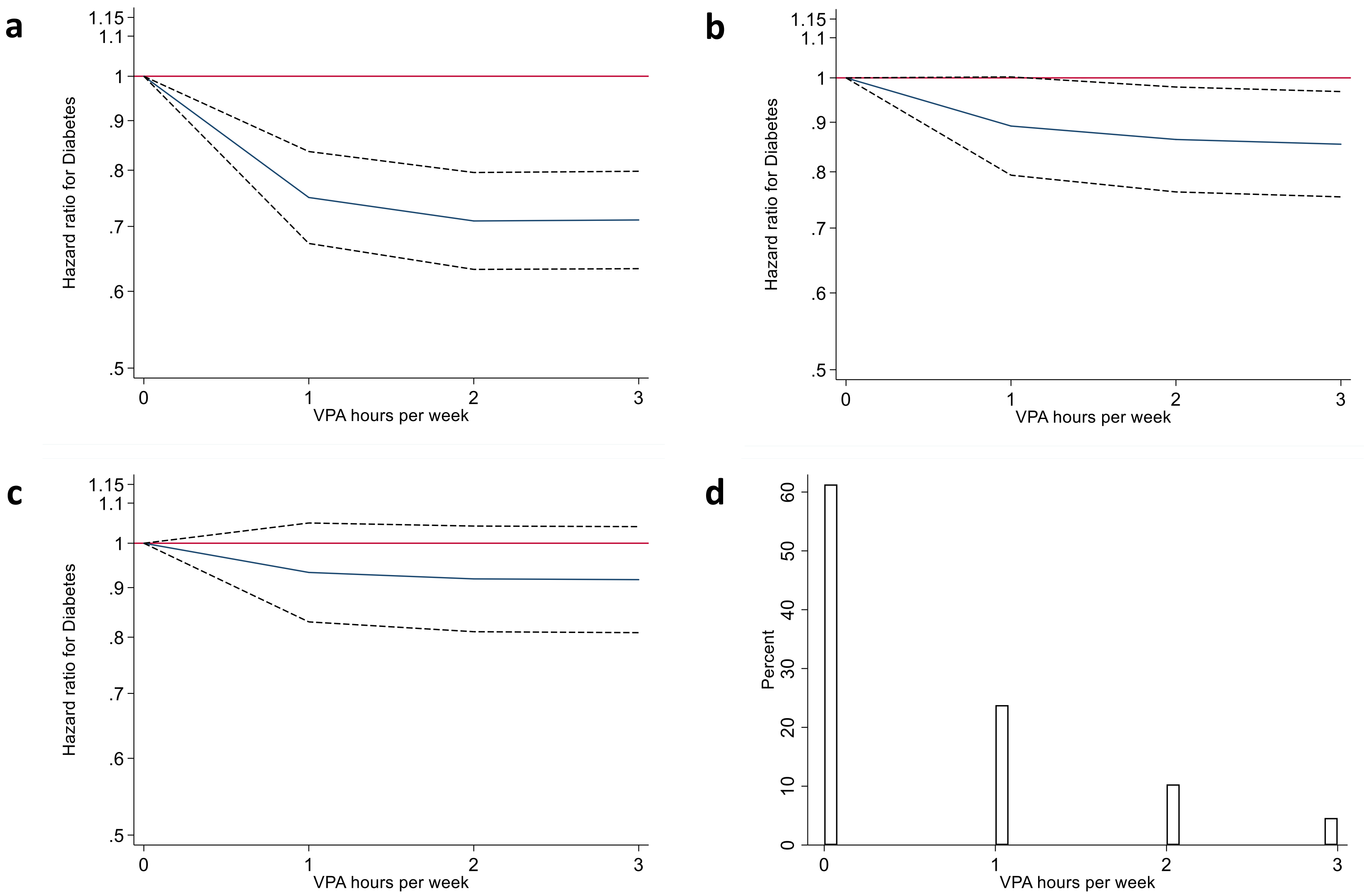

ESM Fig 3: Dose-response relation of vigorous physical activity with risk of incident diabetes. (**a**) crude model; (**b**) adjusted for age (time-scale), sex, ethnicity, education, occupational grade, marital status, smoking status, alcohol consumption, fruits & vegetables intake and MPA; (**c**) additionally adjusted for BMI, hypertension, total cholesterol, CVD drugs, and prevalent CVD; (**d**) population distribution. Blue line represents the HR using restricted cubic spline regression models (3 knots). The reference value (HR=1) is 0 hour/week. Black dash lines are 95 % CI. VPA, vigorous physical activity; MPA moderate physical activity.

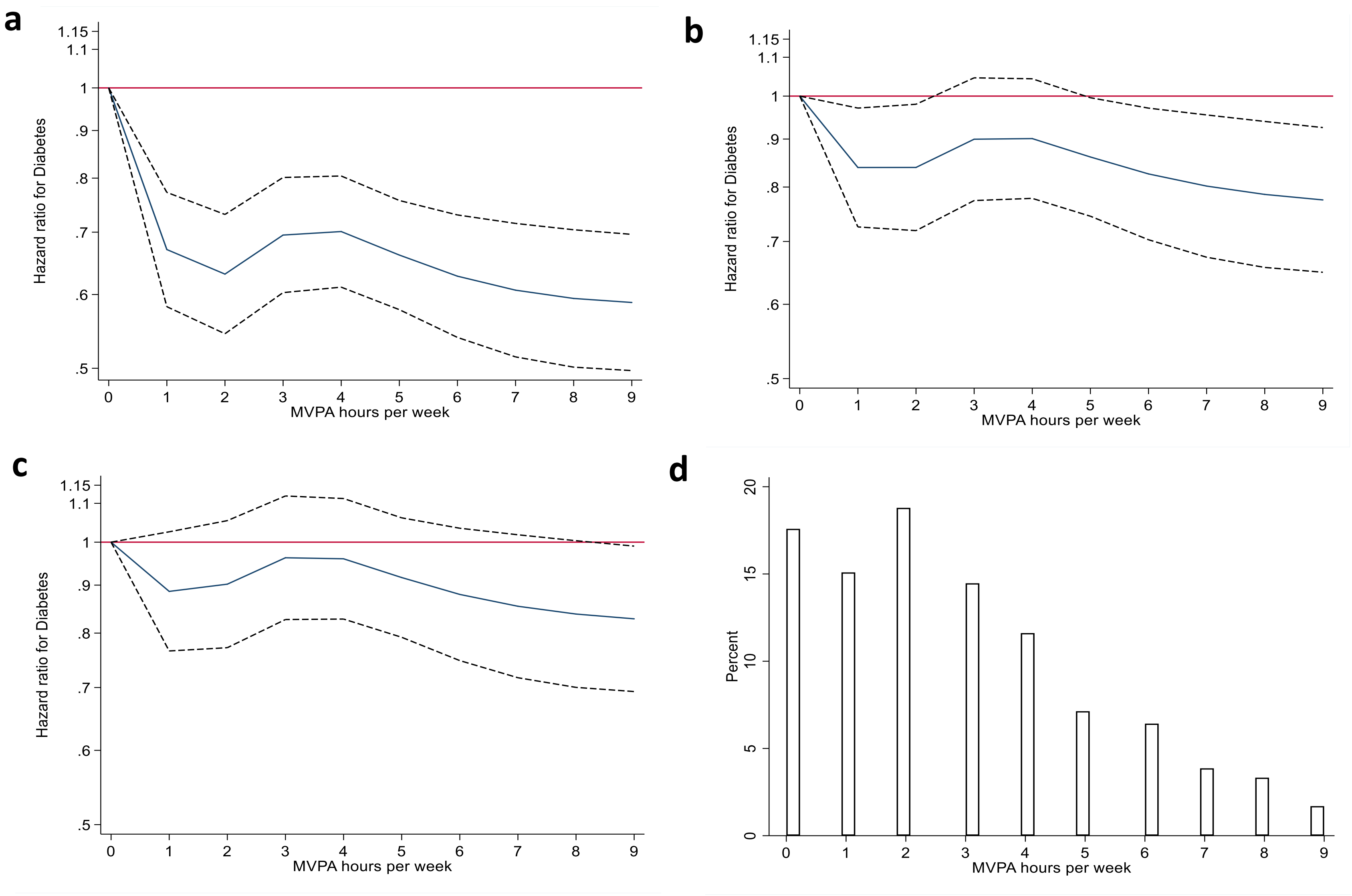

ESM Fig 4: Dose-response relation of moderate-to-vigorous physical activity with risk of incident diabetes. (**a**) crude model; (**b**) adjusted for age (time-scale), sex, ethnicity, education, occupational grade, marital status, smoking status, alcohol consumption, fruits & vegetables intake and MPA; (**c**) additionally adjusted for BMI, hypertension, total cholesterol, CVD drugs, and prevalent CVD; (**d**) population distribution. Blue line represents the HR using restricted cubic spline regression models (5 knots). The reference value (HR=1) is 0 hour/week. Black dash lines are 95 % CI. MVPA, moderate-to-vigorous physical activity.

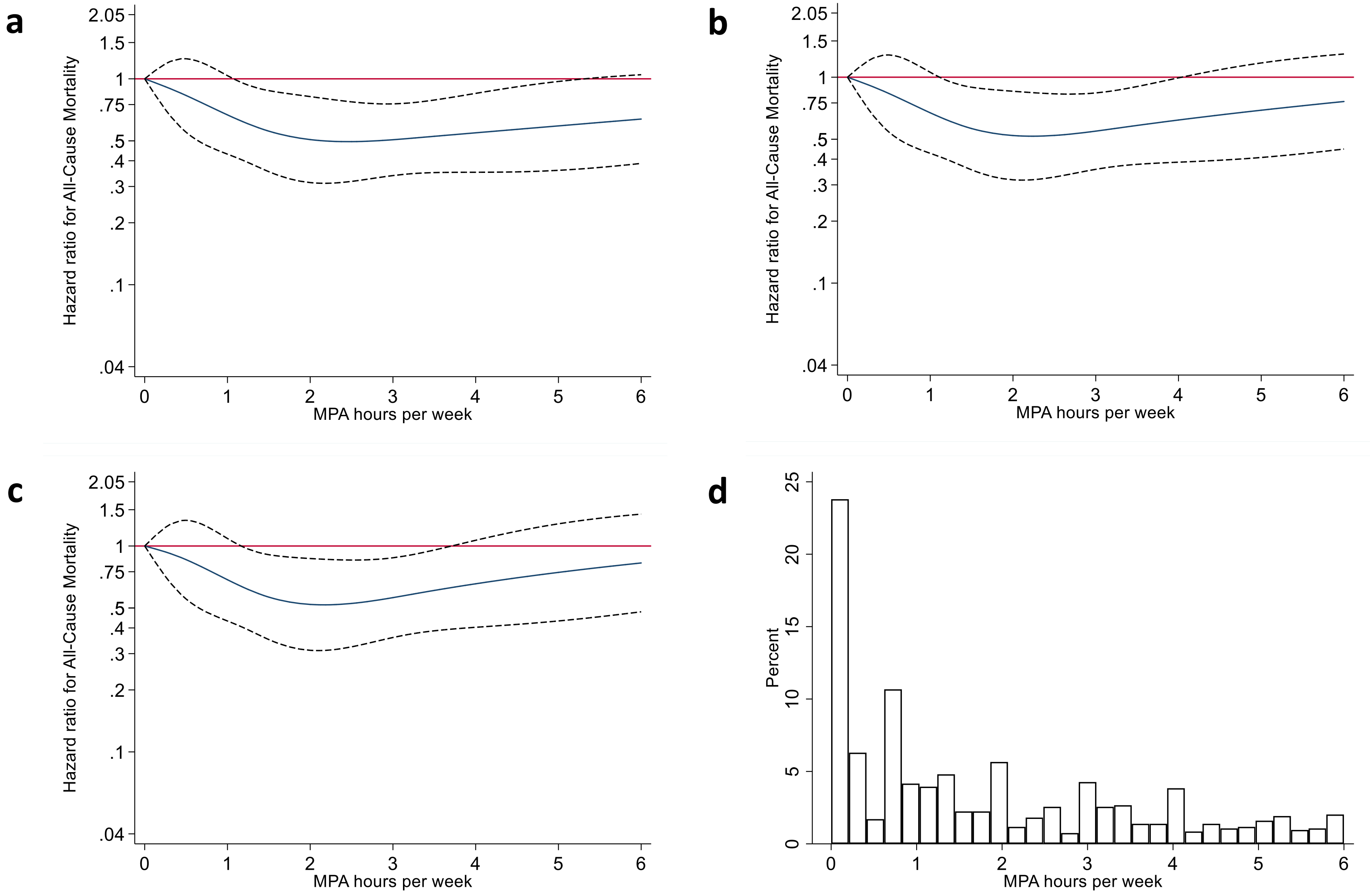

ESM Fig 5: Association of moderate physical activity post-diabetes diagnosis with risk of all-cause mortality. (a) crude model; (b) adjusted for age (time-scale), sex, ethnicity, education, occupational grade, marital status, smoking status, alcohol consumption, fruits & vegetables intake and VPA; (c) additionally adjusted for BMI, hypertension, total cholesterol, SF-36 mental and physical component summary scores, CVD drugs and prevalent CVD; (d) population distribution. Blue line represents the HR using inverse probability weighted restricted cubic spline regression models (5 knots). The reference value (HR=1) is 0 hour/week. Black dash lines are 95 % CI. MPA, moderate physical activity; VPA, vigorous physical activity.

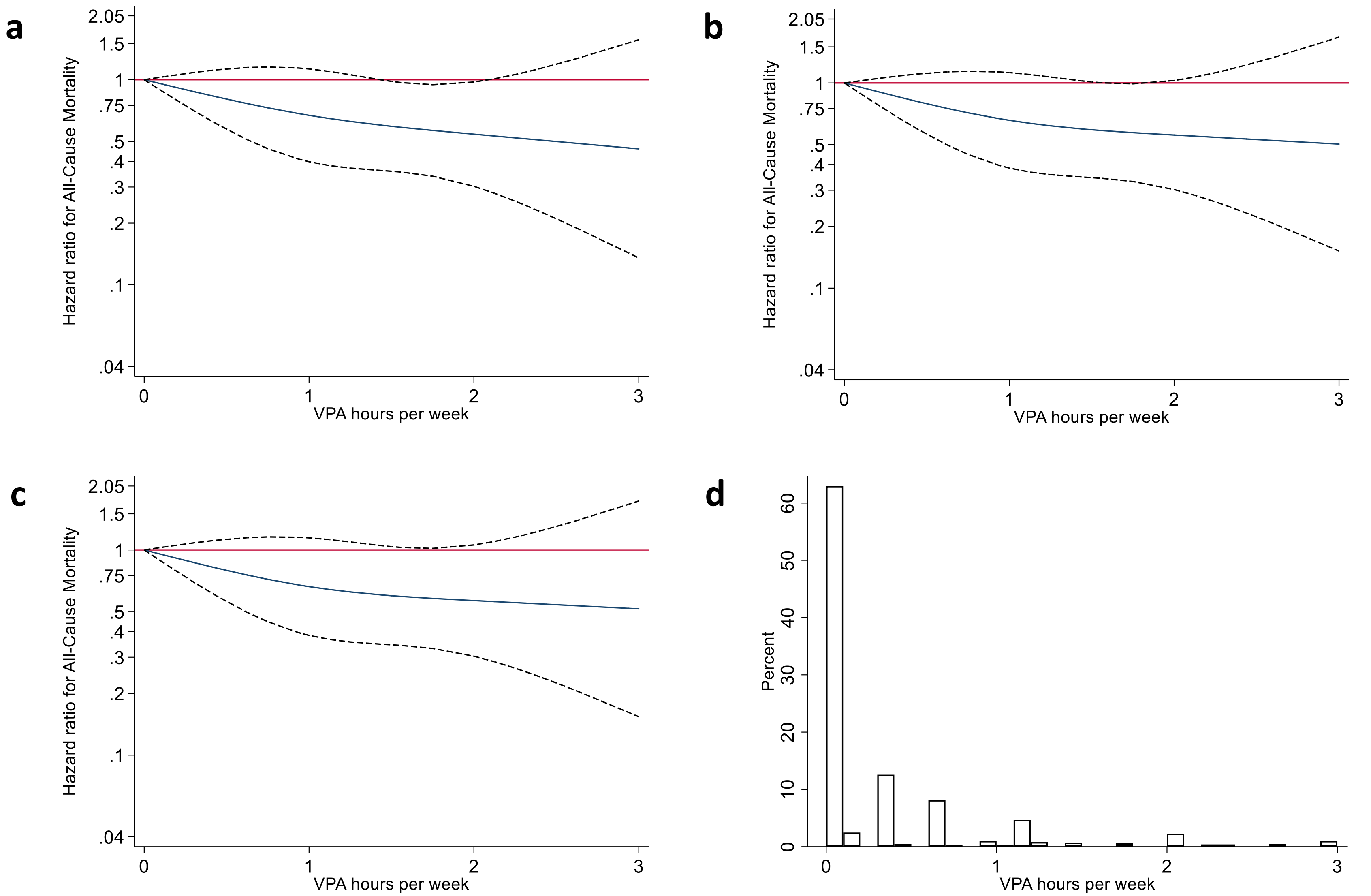

ESM Fig 6: Association of vigorous physical activity post-diabetes diagnosis with risk of all-cause mortality. (a) crude model; (b) adjusted for age (time-scale), sex, ethnicity, education, occupational grade, marital status, smoking status, alcohol consumption, fruits & vegetables intake and MPA; (c) additionally adjusted for BMI, hypertension, total cholesterol, SF-36 mental and physical component summary scores, CVD drugs and prevalent CVD; (d) population distribution. Blue line represents the HR using inverse probability weighted restricted cubic spline regression models (3 knots). The reference value (HR=1) is 0 hour/week. Black dash lines are 95 % CI. VPA, vigorous physical activity; MPA, moderate physical activity.

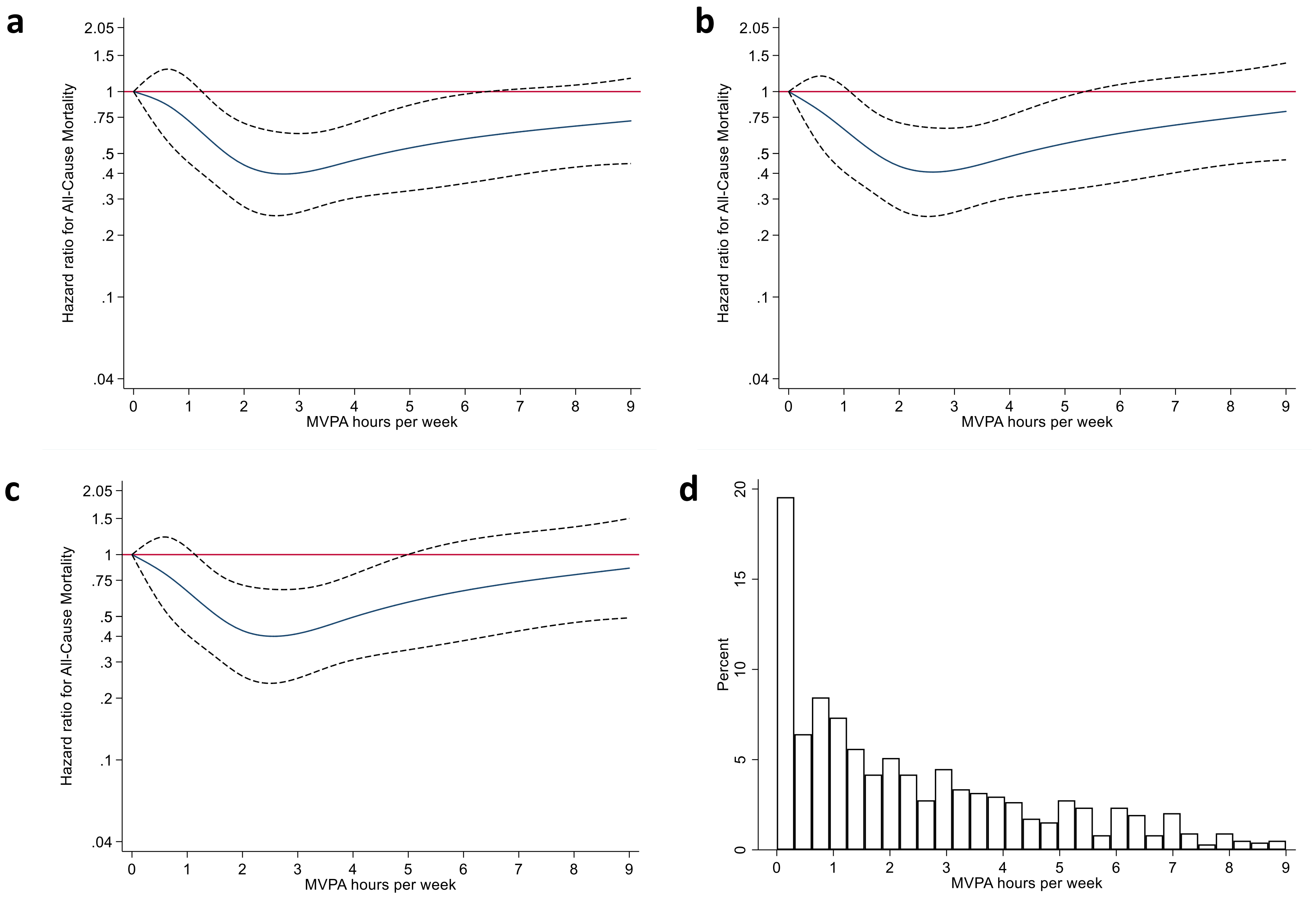

ESM Fig 7: Association of moderate-to-vigorous physical activity post-diabetes diagnosis with risk of all-cause mortality. (a) crude model; (b) adjusted for age (time-scale), sex, ethnicity, education, occupational grade, marital status, smoking status, alcohol consumption, fruits & vegetables intake; (c) additionally adjusted for BMI, hypertension, total cholesterol, SF-36 mental and physical component summary scores, CVD drugs and prevalent CVD; (d) population distribution. Blue line represents the HR using inverse probability weighted restricted cubic spline regression models (5 knots). The reference value (HR=1) is 0 hour/week. Black dash lines are 95 % CI. MVPA, moderate-to-vigorous physical activity.

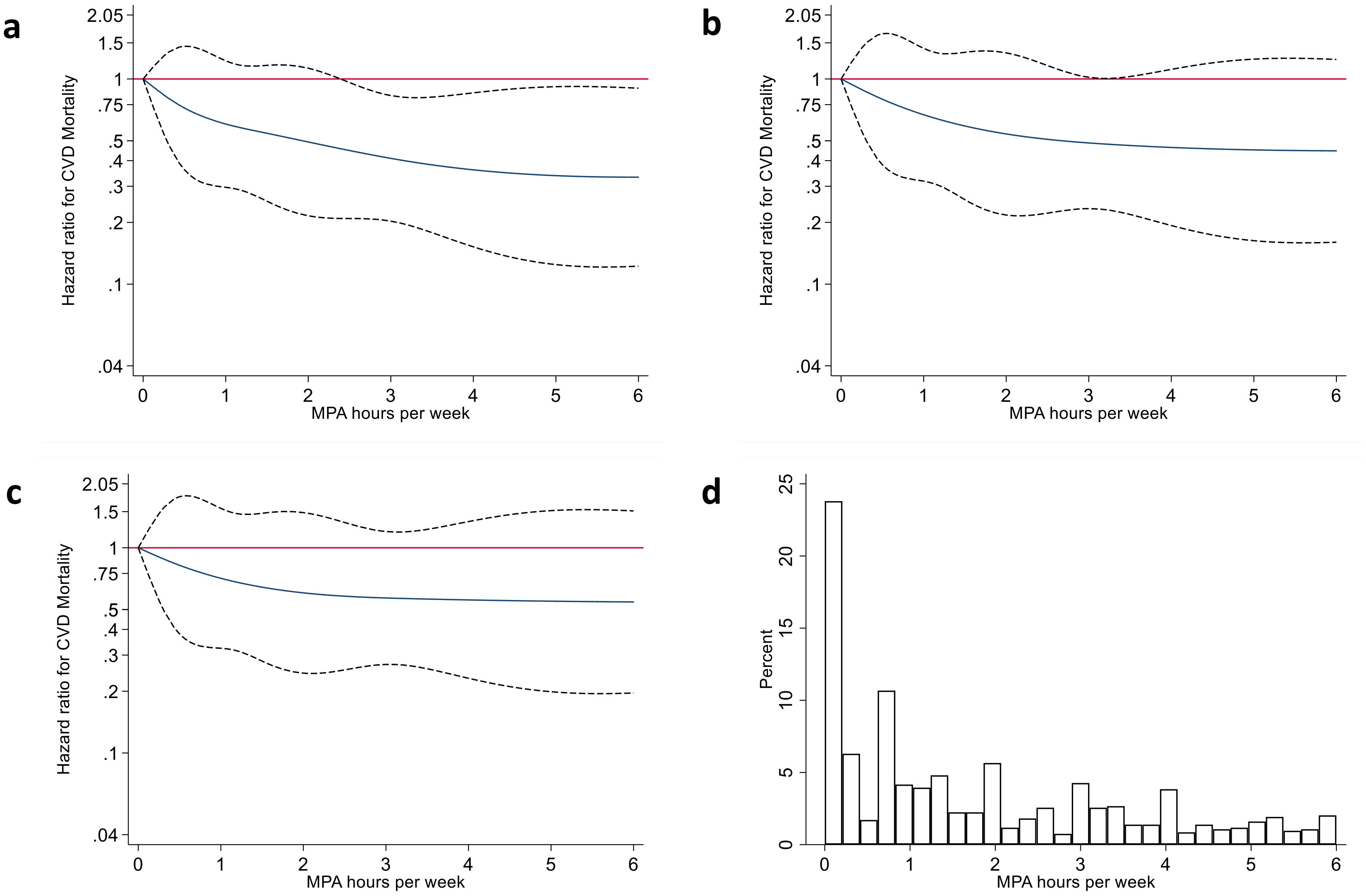

ESM Fig 8: Association of moderate physical activity post-diabetes diagnosis with risk of CVD mortality. (a) crude model; (b) adjusted for age (time-scale), sex, ethnicity, education, occupational grade, marital status, smoking status, alcohol consumption, fruits & vegetables intake and VPA; (c) additionally adjusted for BMI, hypertension, total cholesterol, SF-36 mental and physical component summary scores, CVD drugs and prevalent CVD; (d) population distribution. Blue line represents the HR using inverse probability weighted restricted cubic spline regression models (5 knots). The reference value (HR=1) is 0 hour/week. Black dash lines are 95 % CI. MPA, moderate physical activity; VPA, vigorous physical activity.

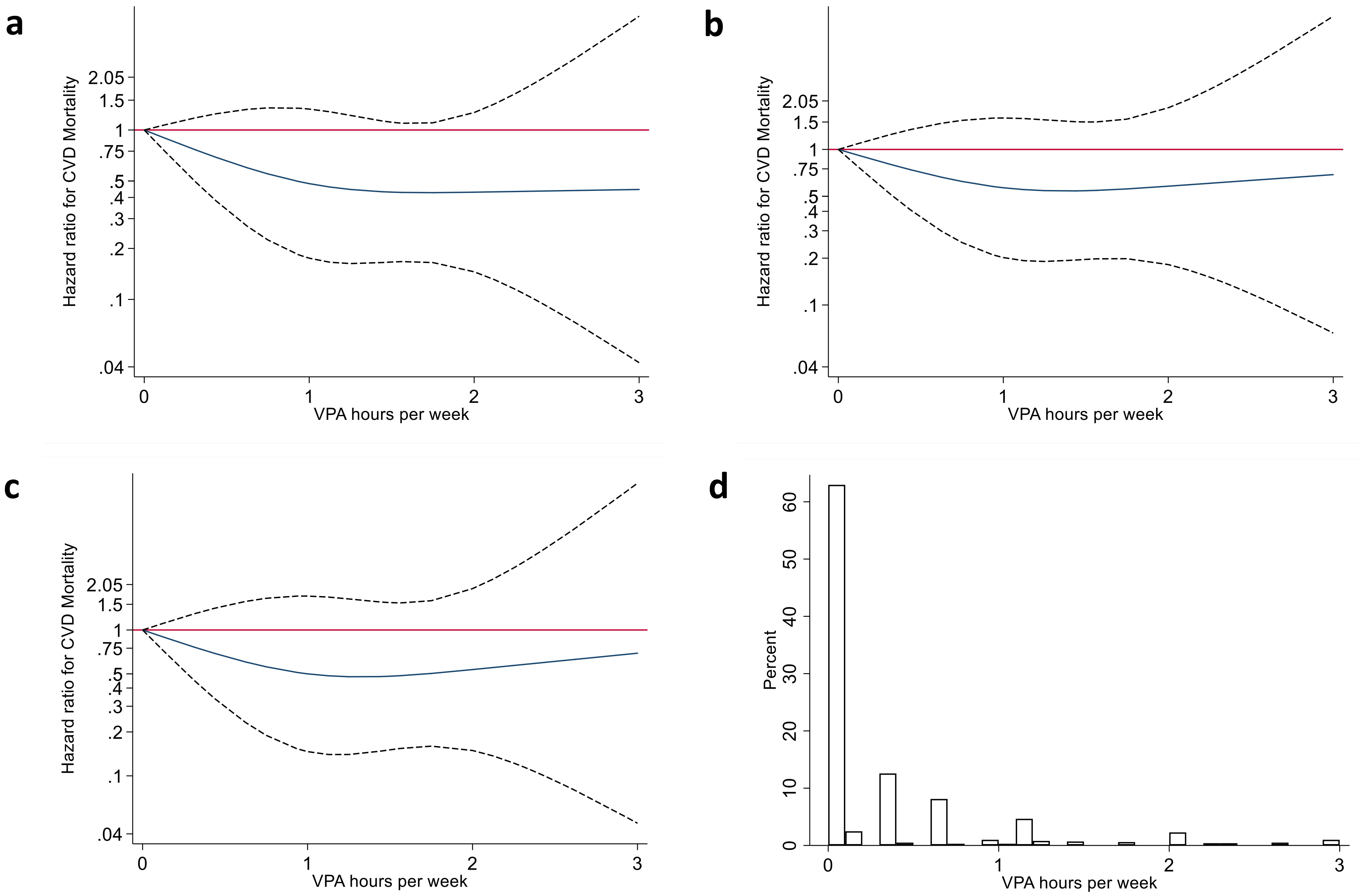

ESM Fig 9: Association of vigorous physical activity post-diabetes diagnosis with CVD mortality. (a) crude model; (b) adjusted for age (time-scale), sex, ethnicity, education, occupational grade, marital status, smoking status, alcohol consumption, fruits & vegetables intake and MPA; (c) additionally adjusted for BMI, hypertension, total cholesterol, SF-36 mental and physical component summary scores, CVD drugs and prevalent CVD; (d) population distribution. Blue line represents the HR using inverse probability weighted restricted cubic spline regression models (3 knots). The reference value (HR=1) is 0 hour/week. Black dash lines are 95 % CI. VPA, vigorous physical activity; MPA, moderate physical activity.

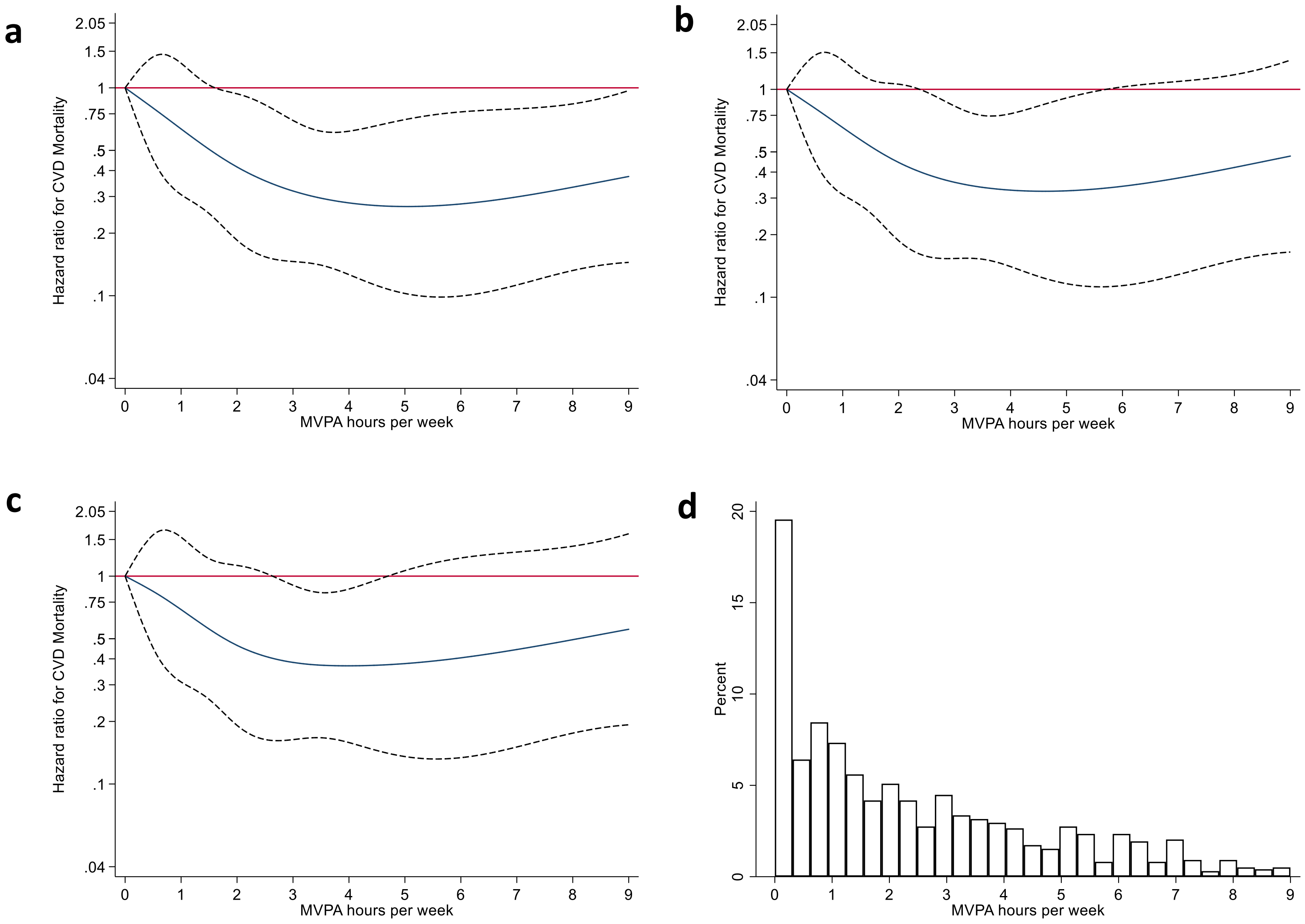

ESM Fig 10: Association of moderate-to-vigorous physical activity post-diabetes diagnosis with CVD mortality. (a) crude model; (b) adjusted for age (time-scale), sex, ethnicity, education, occupational grade, marital status, smoking status, alcohol consumption and fruits & vegetables intake; (c) additionally adjusted for BMI, hypertension, total cholesterol, SF-36 mental and physical component summary scores, CVD drugs and prevalent CVD; (d) population distribution. Blue line represents the HR using inverse probability weighted restricted cubic spline regression models (5 knots). The reference value (HR=1) is 0 hour/week. Black dash lines are 95 % CI. MVPA, moderate-to-vigorous physical activity.
